# Recovery from suicidality in youth aged 6 to 25: a review of 50 quantitative and qualitative studies

**DOI:** 10.1007/s00787-026-02991-y

**Published:** 2026-04-11

**Authors:** Mandy Gijzen, Diana van Bergen, Maria Esanu, Bertus F. Jeronimus

**Affiliations:** 1 Department of Pedagogics and Education, Faculty of Social and Behavioural Sciences, Groningen University, Groningen, The Netherlands; 2 Department of Developmental Psychology, Faculty of Behavioural and Social Sciences, Groningen University, Groningen, The Netherlands

**Keywords:** Systematic review, Lived experience, Self-harm, Suicidal ideation, Social support, Recovery

## Abstract

Suicide is a leading global cause of death among youth, with suicidality encompassing ideation, self-harm, and suicides. Despite its severity, a third of suicidal and self-harming adolescents recover before adulthood, highlighting the importance of identifying recovery facilitators for effective interventions. This scoping review was aimed to synthesize how recovery from suicidality is defined and experienced by young people aged 0 to 25, and to identify the psychosocial and contextual factors that facilitate or hinder recovery. We conducted a scoping review of 50 qualitative and quantitative studies involving 28,299 participants. Studies were identified in Medline, PsycInfo, SocINDEX, and CINAHL databases. Quality was assessed using CASP and QUIPS tools. We extracted recovery-related themes and categorized them into thirteen thematic domains. Social support, coping skills, and personal acceptance were most endorsed. Recovery was described both as symptom remission and as a personal transformative process involving identity development, emotional regulation, and meaning-making. Recovery was shaped by both internal capacities and external support, including therapeutic relationships, peer connections, and access to identity-affirming or culturally responsive care. Youth emphasized the long-term, personal nature of recovery, extending beyond suicidality to include positive mental health, self-worth, and purpose. Recovery from youth suicidality is multidimensional, and shaped by developmental, relational, and sociocultural contexts. Effective prevention and intervention must go beyond risk reduction to foster protective and promotive factors that empower young people. These findings support the need for more holistic, person-centered approaches to youth mental health and suicidality.

**Supplementary Information:**

The online version contains supplementary material available at 10.1007/s00787-026-02991-y.

## Introduction

Suicide in youth (0–25 year olds) is a major global public health concern. While suicide in children (> 10 year olds) is relatively rare, it ranks as the third leading cause of death among adolescents aged 10–14 years, rising to the second leading cause in older adolescents (aged 15–19) and young adults (aged 20–24) [1, 2]. Non-fatal self-harm in youth is far more common, often repeated, and strongly associated with suicide [3–7]. In the present review, we adopt the definition of self-harm commonly used in UK and European research, in which self-harm refers to any act of intentional self-injury or self-poisoning, irrespective of the degree of suicidal intent (e.g., Hawton et al. 2003, 2012 [4, 8]). Within this framework, suicidal ideation refers to thoughts about ending one’s life, whereas self-harm captures behavioral expressions of self-injury that may occur with or without an intention to die. We acknowledge that (a) other research traditions distinguish non-suicidal self-injury (NSSI) from suicidal behavior (e.g., Nock 2006 [9]); and (b) that self-harm often functions as a coping strategy to manage distress rather than an act oriented toward death [10].

We build on the co-occurrence of non-suicidal self-injury and suicide attempts in youth, the fluidity of self-harm motivations, and the fact that many self-harm acts cannot be conclusively classified as either NSSI or suicide attempts. Following the Hawton tradition in suicidology, we therefore conceptualize suicidality as encompassing suicidal ideation and self-harm behaviors [11]. SI often precedes SH, and self-harm episodes typically begin in early adolescence, peaking around age 16 [12–15]. During adolescence, self-harm is particularly closely linked to suicide, which remains one of the leading causes of mortality in this age group. Community-based data suggest that for each adolescent who dies by suicide, there are hundreds of SH episodes, highlighting the critical importance of addressing both ideation and self-harm early [13, 15]. Population-based data indicate that 14–23% of youth experience SI, with 8–9% forming a suicide plan [16–18], while 5–16% engage in SH [13, 16–18]. Self-harm is common, frequently repeated, and often occurs in the community, far exceeding the number of fatal suicides [3, 13, 15]. Thus, adolescent SI and SH often co-occur in adolescents, reflecting the shared underlying distress and risk. Among adolescents who self-harm, approximately two-thirds report at least some desire to die during episodes [19], and population-based longitudinal data indicate that youth who experience both SI and non-suicidal self-harm have a markedly higher likelihood of subsequent suicidal behaviors compared with peers without these experiences [20]. This co-occurrence underscores the importance of considering SI and SH as interconnected phenomena in youth.

Even though suicidality signal the hopelessness and despair these youth experience in their lives, prospective studies show that one third of suicidal and self-harming adolescents actually recover before they reach adulthood [21–26]. Despite extensive research on risk factors for suicidality, *recovery* from SI and SH has received comparatively less attention [27–30]. Most theoretical models and longitudinal studies focus on early warning signs, symptoms, or factors that predict persistent suicidality. However, building and strengthening unique recovery factors may yield a stronger reduction in youth suicidal behavior than merely reducing risk factors [31]. Evidence suggests that adolescent risk factors alone cannot adequately distinguish youth who recover from SI and SH from those with persistent distress or from peers who never experience suicidality [32]. Consequently, understanding factors that actively foster recovery is essential for advancing prevention and intervention strategies [27, 30, 33]. Mental recovery is increasingly conceptualized as a multifaceted process, encompassing existential dimensions (e.g., agency, self-efficacy, meaning), social dimensions (e.g., interpersonal connections), physical factors (e.g., exercise, substance use), and functional components (e.g., educational or occupational attainment), alongside symptom reduction [34–38]. Recovery should be viewed as a continuum, influenced by age, developmental stage, and social and cultural context [39–42].

Four prior qualitative reviews of recovery from suicidality have identified key protective factors, including supportive social connections, meaningful activities, coping strategies, and a sense of belonging [43–45]. While these reviews have advanced understanding of lived experiences of recovery, they have largely synthesized qualitative evidence and offered limited integration with quantitative findings or explicit consideration of how differing definitions of recovery shape study inclusion and interpretation. Also, these reviews were limited by being focused on older adults or post-acute samples. These findings nevertheless align with recent theoretical models of recovery, including the COURAGE framework [46], which conceptualizes recovery as a set of seven interrelated, non-linear processes that may occur in any sequence: choosing life (a cognitive and emotional decision to engage with life), optimizing identity (developing self-confidence and a coherent life story), understanding oneself (reflecting on patterns of distress and personal strengths), rediscovering meaning in life (finding purpose through goals, spirituality, or community), acceptance (acknowledging internal pain and receiving social validation), growing connectedness (building meaningful relationships and support networks), and empowerment (developing skills, agency, and confidence to navigate life and seek help) [46].

Recovery processes also intersect with established theoretical models of suicide. The Interpersonal Theory of Suicide (IPTS) highlights perceived burdensomeness and thwarted belongingness as central mechanisms underlying suicidal desire [47], which align with the COURAGE that could help people to overcome these factors: processes of connectedness and acceptance. Similarly, classical theories of suicide, such as Shneidman’s concept of *psychache* (intense psychological pain due to unmet needs) [48], Baumeister’s *escape theory* (suicide as escape from aversive self-perception) [49], and Williams’ *cry-of-pain model* (suicide as a response to defeat, entrapment, and lack of rescue) [50], emphasize that suicidal behavior often arises from overwhelming distress and a lack of coping resources. These theories converge conceptually with the COURAGE framework by highlighting the role of meaning-making, connectedness, agency, and hope in fostering recovery.

The present review extends prior work by integrating both quantitative and qualitative studies on recovery from suicidality in youth aged 0–25 years. We focus on SI and SH as core phenomena, in line with contemporary European conceptualizations where self-harm encompasses behaviors with or without suicidal intent [8, 15]. By examining how recovery is defined and operationalized, and by considering social, existential, and functional dimensions, this review aims to provide a comprehensive foundation for theory, prevention, and clinical intervention. Specifically, this review addresses three interrelated aims: (1) to clarify how recovery is defined by youth, (2) to summarize observed recovery outcomes (how researchers operationalize recovery), and (3) to identify the factors and processes that facilitate recovery (recovery conditions). Explicitly distinguishing recovery conditions from outcomes helps ensure conceptual clarity and guides the synthesis of evidence.

## Method

We implemented a scoping review strategy [51] and pre-registered our aims at the Open Science Framework [52]. The review followed the PRISMA-ScR (Preferred Reporting Items for Systematic Reviews and Meta-Analyses extension for Scoping Reviews) checklist to ensure comprehensive and transparent reporting of key items [53]. Although our review was initially structured using Arksey and O’Malley’s stages, our procedures are in accordance with contemporary scoping review guidance from the Joanna Briggs Institute [54–57], including the use of predefined objectives and eligibility criteria, systematic search and study selection procedures, structured data charting, and narrative synthesis.

We included empirical studies of (1) children, adolescents, or young adults aged 0–25 who reported suicidality and (2) recovered from suicidality. We defined suicidality both as feelings and thoughts about suicidality (suicidal ideation) and self-harm (self-injury, suicide attempts, acts in preparation of suicide). Recovery was operationalized as qualitative narratives (or accounts), quantitative measures or reports of overcoming suicidality, of recovery during treatment (both psychological and pharmacological treatment), and experiences of patients/clients/people with suicidality that overcame their suicidality. In line with our review aims, we distinguished between conceptualizations of recovery outcomes (i.e. how recovery from suicidality is defined or described and indicates improvement such as reduced suicidal ideation, cessation of self-harm, or improved functioning), versus recovery conditions (i.e. factors, processes, or contexts reported to facilitate or support recovery). We focused primarily on identifying recovery conditions and how recovery was conceptualized in the literature, but because many primary studies did not consistently differentiate recovery conditions from recovery outcomes, we extracted and coded both. The review primarily sought to synthesize first-person accounts of recovery from suicidality and self-harm. However, studies explicitly examining parents’ or mental health professionals’ perspectives on recovery processes were also included, as these offer complementary insight into how recovery is conceptualized and supported in practice. This multi-perspectival approach allowed integration of lived-experience and clinical viewpoints while maintaining a primary focus on young people’s recovery processes. We did not include studies that examined the effects of clinical or pharmaceutical treatment of suicide, nor did we cover accounts of how a wound or physical damage/injury due to self-harm was treated. Also, excluded stories of participants bereaved by suicide, as these stories tend to focus on grief processes or perspectives of ‘causes’ of suicide.

We focused on individuals aged 0–25 years to capture recovery processes in childhood, adolescence, and emerging adulthood as these are periods marked by heightened vulnerability to self-harm and suicidality, rapid social and identity development, and evolving emotion-regulation capacities [58–60]. Recovery trajectories may extend beyond these ages; consequently, some studies included adults reflecting retrospectively on earlier suicidal experiences. In such cases, we extracted the age at the time of suicidality to ensure that included data reflected the relevant developmental period. We acknowledge that retrospective accounts may differ from contemporaneous reports, and we address this interpretive consideration in the Discussion.

### Search strategy

We identified articles published before February 6th, 2025, in the compendium indexed in Medline, PsycInfo, SocINDEX and CINAHL, using search strings related to (1) suicidality, (2) recovery and (3) young people provided in Supplementary Material Table S1. We screened reference lists of included articles to identify potentially missed studies.

### Study selection

After the removal of duplicate articles two reviewers scanned all titles and abstracts according to our inclusion criteria (MG, DB, BJ, AS, ME, MK, NO), who discussed discrepancies until consensus was derived. The Rayyan program was used for title and abstract selection [61]. The inter-rater inclusion agreement was ~ 84% between each set of reviewers, which is generally deemed acceptable [62]. Of the remaining articles, full-texts were retrieved and reviewed by the first author, who also extracted the data. Papers in a language other than English or Dutch were translated using Google to access the full-texts (following recommendations by [63]), which applied to one Spanish paper [64]. Finally, three PhD dissertations met our inclusion criteria and were included in the data extraction, synthesis, and quality appraisal process. Although theses have not undergone the same rigorous reviewing process as papers in scientific journals, there was an expert reading committee, and because dissertations met al.l other inclusion criteria, and passed our quality appraisal, we included their results (see Table 2).

### Data synthesis

For qualitative and mixed-methods studies, we synthesized data on author-reported themes and, where available, illustrative participant quotations reflecting lived experience. All extracted qualitative findings were initially coded by identifying themes within larger text segments, generating descriptive codes that captured reported recovery-related processes. From quantitative papers we extracted the factors that were related to fostered the recovery process from suicidality. We applied an inductive thematic synthesis approach [65]. Codes were iteratively grouped into higher-order categories through constant comparison across studies. Coding was performed by the first author, and discussed with DB and BJ. The resulting categories were then organized into overarching thematic domains aligned with the review aims. We identified 95 codes from the included papers (see Supplementary Materials) which were subsequently grouped into 13 thematic categories by the first author (using feedback by DB/BJ), which we organized using Atlas.ti.

### Reflexivity statement

The review team’s disciplinary backgrounds in clinical psychology, suicidology, and recovery-oriented mental health research informed our interpretation of the data. We approached recovery as a dynamic, person-centered process extending beyond symptom reduction. Reflexive discussions were held throughout synthesis to consider how these assumptions might shape coding and theme development.

### Quality assessment

Qualitative study quality was scored with the Critical Appraisals Skills Programme (CASP; [66]) and quantitative study quality with the Quality In Prognosis Studies tool (QUIPS; [67]), each completed by at least two reviewers independently (MG/DB/BJ), and discrepancies were discussed until consensus was reached.

## Results

**Fig. 1:**
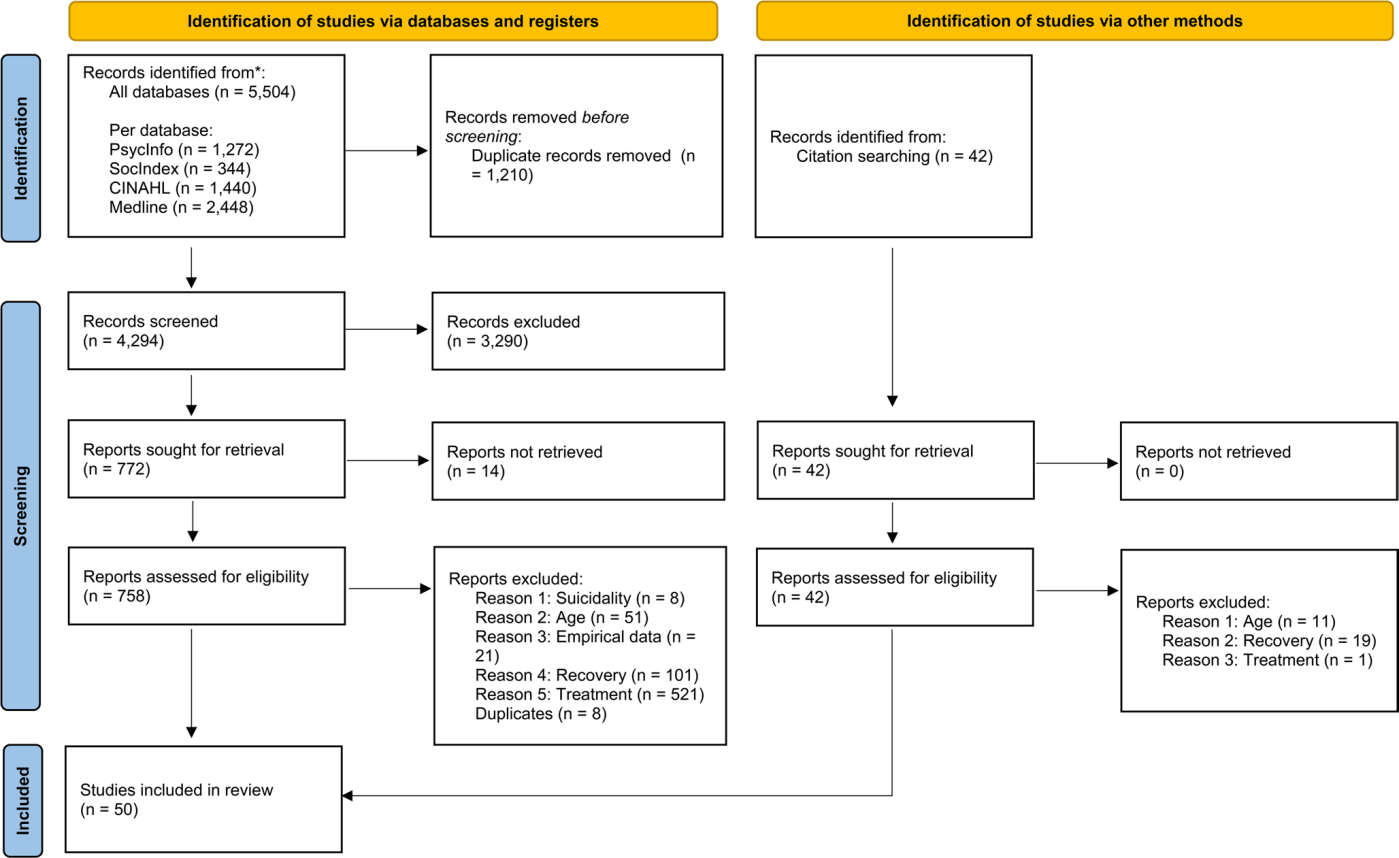
Flow chart of included studies. *Note*. Flowchart based on Page et al. (2021) [146]. Exclusion criteria: Suicidality – No suicidality was mentioned. Age: Mean age was > 25. Empirical data: No empirical was collected. Recovery: No factors related to recovery were described in paper. Treatment: Study focused exclusively on treatment or intervention effects

We identified 4,294 articles, after removal of 1,210 duplicates, of which 772 articles were selected using title and abstract screening, see the Flow Chart in Fig. 1. The backward snowballing strategy using previous reviews resulted in another nine articles. Full texts were perused and 50 papers were included in the current review spanning 28,299 participants, see Table 1 [24, 64, 68–114], and most papers were excluded because they focused exclusively on treatment or intervention effects (*n* = 521) or not on recovery (*n* = 101). We included three PhD dissertations (see method section).

**Table 1:** Key characteristics and recovery factors of included studies

First Author Year	N, ♀%	Participants	ISO	Mean age and age Range in years	Study type	Time *T*_0_-*T*_1_
Bautista 2023	7 NR 5 SA survivors 60% 7 MH profs NR	SA survivors MH profs	PH	SA survivors: M = 23·2 R = 21–27	Intv (SS)	NR
Bennett 2002	30 NR	R = 15–24 yrs., ≥ 1 SA at ED	NZ	M = NR R = 15–24	Intv (SS)	*T*_0_ = < 2 wk. post- SA,* T*_1_ = + 9 mo
Bergmans 2009	16 88%	< 25 yrs.; ≥ 1 intervention cycle for repeated SA	CA	*T*_0_ = 22·3 *T*_1_ = 25·8 R = 18–25	Intv	R = 0·5–6 yrs., *T*_SA1_: M_age_ = 15·1
Bostik 2007	50 82%	STBs 13–19 y.o; No STBS > 6 Mo; STBs < 3 yrs	CA	M = 21·9 R = 13–26	Intv. (SS)	NR
Buelens 2023	2161 53·9%	Adolescents	BE	M = 15 R = 10–21	Q_s_	T_1_ = 1 yr T_2_ = 2 yrs
Buser 2014	12 83%	past NSSI^4^	US	M = 21.5 R = 19–27	Intv. (SS)	N/A
Claréus 2021	11 46%	Disc. repetitive NSSI > 5 at *T*_3_ but not *T*_0_-*T*_1_	SE	M = NR R = 25–26	Intv. (SS)	9–10 yrs
Claréus 2023	557 59·2%	Public school students	SE	*T*_0_ = 13·7 *T*_1_ = 14·8 *T*_2_ = 25·3 R = NR	Q_s_	*T*_1_ = 1 yr *T*_2_ = 10 yrs
Everall 2005	1 100%	♀SI R = 14–18 yrs	CA	13	Interview (SS)	2 yrs
Everall 2006	13 100%	STBs SR R = 15–24 yrs. & < 3 yrs. & ≥ 6 mo. < *T*_0_	CA	M = 21·4 R = 17–26	Intv. (SS)	≥ 6 mo
Fenaughty 2003	8 0%	Queer♂ aged > 26 yrs ≥ 1 SA	NZ	M = 19.9 R = 18–23	Intv. (SS)	NR
Gelinas 2013	54 85%	Undergraduate students	CA	M = 20·9 R = 18–35	Online Q_s_ with open-ended items	NR
Goncalves 2023	385 85·2%	College students	PT	M = 20·7 R = 18–35	Qs	N/A
Gulbas 2019	17 100%	Latina teens, typically low-income NY households	US	M = 16 R = 14–18	Intv. (SS)	6 + 12 mo. after SA
Hasking 2024	733 82·5%	Anyone with a history of NSSI	CA, AU	M = 24·5 R = 14–62	Qs	N/A
Holliday 2015	6 83·3%	≥ 1 SA past 6 mo. ED adm	US	R = 15–19	Intv	NR
Keefner 2020	12 100%	Young adults R = 18–25 yrs	US & CA	NR (NR) median age = 23	Intv. (SS)	NR
Kelada 2018a	48 NR	Adolescents between 12–18 yrs old	AU	M = 14·5, R = 12–18	Q_s_	NR
Kelada 2018b	98 NR	Youngsters	AU, BE, US	AU M = 15·2 R = 12–18 BE M = 17·3 R = 17–19 US M = 20·2 R = 15–26 Overall R = 12–25	Youngsters AU + BE SR Q_s_ but US intv	NR
Knowles et 2022	23 48% NZ: 13 UK: 10	Youth with lived exp of SH or indirect exp. of SH/suicide as friend or family	NZ, UK	NZ M = NR R = 16–23 UK M = NR R = 18–23	Codesign workshops	NR
Kolar 2012	10 50%	Street-involved youth (SIY)	CA	M = 23·2 R = 19–26	Intv	NR
Kwok 2019	909 37%	Adolescents Grade 8–9	HK	*T*_0_ = 13·9 R = 12–18	Q_s_	12 mo
Lewis 2019	232 78%	Emerging adults	CA	M = 19 R = NR	Q_s_ with open-ended items	NR
Meheli 2022	229 79%	University students	CA	M = 19 R = NR	Q_s_ with open-ended items	NR
Meheli 2023	30 66·7%	People with history of NSSI	IN	M = 20·4 R = 14–32	Q_s_	NR
Muehlenkamp 2018	644 NR	University students who engaged in NSSI past yr	US	18.9 (NR)	Q_s_	NR
Norton 2011	1 100%	16-y.o. Caucasian♀	US	16 (N/A)	Case study	NR
Ortin 2019	1228 48%	PR adolescents	US	NR (10–13)	Q_s_	NR
Özen-Dursun 2023	11 45·5%	Asians living in the UK with history of SH	UK	M = 25·9 R = 19–26	Interview (SS)	NR
Puotiniemi 2004	1 100%	Girl and mom in treatment	NR	NR	Intv	NR
Real 2025	661 48·3%	LGBTQ + youth 15–21 yrs	US	M = 18·5 R = 15–21	Qs	*T*_1-4_ each + 9 mo
Redmond 2020	7 71%	Mental health support workers	UK	M = NR R = 21–45 (who treat 15–20 yrs.♀)	Intv. (SS)	NR
Rissanen 2013	347 91·9%	Adolescents 13–18 yrs	FI	M = NR R = 13–18	Q_s_ with open-ended items	NR
Shaw 2006	5 100%	♀College students who had engaged in treatment for SH	US	M = 20·2 R = 19–21	Intv	T since last SH R = 10 mo. to 5 yrs
Szlyk 2021	3 33%	Students enrolled in alternative high school	US	M = 17·7 R = 17–18	Intv.(SS)	NR
Tampus-Siena 2023	10 (5 dyads) 20%	College student + their caregiver	PH	M = 22·2 R = 20–25 Caregivers M = 50·8 R = 45–56	Intv	NR
Teismann 2016	1389 100%	Women between 18–24	DE	M = 20·7 R = 18–24	Q_s_	17 mo
Tomicic 2021	30 57% (8 gay♂ 10 gay♀ 5 bi♀ 5 tr♂ 2 tr♀)	LGBT youth between 18–26	CL	M = 21·6 R = 18–26	Interview (SS)	5–13 yrs. (STBs age 13–21 yrs.)
Tong 2022	15,170 NR	Nationally representative sample of US adolescents, grades 7–12	US	*T*_0_ = 16 (12–21) *T*_2_ = 22 (18–28)	Q_s_	6–7 yrs
Tordoff 2022	104 26%	TNB youth	US	M = 15·8 SD = 1·6 R = 13–20	Q_s_	3, 6, 12 mo
Wadman et al. (2017)	6 NR	19–21 y.o	UK	M = NR R = 19–21	Intv. (SS)	SH past 6 mo
Wagner et al. (2000)	100 49%	Patients at the University of Texas- Medical Branch in Galveston	US	M = 13·4 R = 7–17	Q_s_ with open-ended items	7.98 days (SD = 2·92, R = 3–24)
Wang 2023	1518 49·6%	Junior high school students	CN	M = 13.6 R = 11–15	Q_s_	1 yr
Wang 2024	12 58·3%	Youth who survived deliberate self-poisoning	CN	M = 21·1 R = 27–26	Intv. (SS)	< 12 mo.: 5 PPs 12–24 mo.: 3 PPs > 24 mo.: 4 PPs since SA
Whitlock 2015	836 78·3%	Students > 5 instances of NSSI	US	M = 21·3 R = NR	Q_s_ with open-ended questions	N/A
Zeller 2015	64 26%	Exposed to the Mount Carmel Forest Fire Disaster	IL	M = 17·5 R = 15–19	Q_s_	*T*_0_ = ≤ 30-days post-traumatic event *T*_1_ = + 3 Mo *T*_2_ = + 6 Mo
Zortea 2019	9 56%	≥ 1 SA	UK	M = 24·5 R = 20–30 *T*_SI_ = 15·7 R = 11–22 *T*_SA_ = 17·7 R = 13–25	Intv. (SS)	NR
Davis 2018	6 100%	Cisgender lesbians with no SA ≥ 1 10 yrs SA age 12–20	US	M = 41·7 R = 30–61	Intv. (SS)	No SA ≥ 1 10 yrs
Gelinas 2015	10 70%	No SH ≥ 6 mo	CA	M = 19 R = 18–24	Intv. (SS)	Time since last SH from 6 mo. to 6 yrs M = 2·5 yrs
Roberts 2019	32 50%	PP_s_ who experienced SI over adolescence but no SA, who feel they have recovered	US	M = 38, SD = 9·8 R = 21–50	Q_s_ with open-ended items	*T*_SI1_: M_age_ = 14·4

First Author Year	**STBs**	**STBs measure**	**Definition of recovery**	**Recovery measured**	**Recovery by participants**	**Factors related to recovery**
Bautista 2023	SA	NR	Traversing the path, from the state of wanting to die (or brokenness or “burriedness”) to the state of flourishing in life (or sustaining recovery), by which crises, manifested by STBs were overcame through taking recovery actions to allow the needed values to surface and attain recovery	SRAS[147]	Uniquely personal process	1. Social and emotional support 2. Resilience 3. Help-seeking behaviors 4. Keen observation/awareness of suicide recovery behaviors to implement a safety plan **Impeding elements:** 1. Fear to ask for help & lack of social & professional support 2. Family problems 3. Feeling of being a burden 4. Resistance or non-commitment to the treatment process 5. Academic stress 6. History of trauma or abuse
Bennett 2002	SA	Presented at ED	Transitions towards resistance against future STBs	Intv	Process of gradual transition and growth in personal agency and self-responsibility	1. Reconnections with friends and family 2. Change in relations with friends, family and/or environment 3. Help-seeking behaviors 4. Problem solving
Bergmans 2009	SBs	Intv. SR	no SBs post-intervention; 5 (31.3%) reduced SBs	Intv. SR	Gradual over yrs. “to be able to do daily tasks”; “more comfortable with myself”; “living a life that is really mine by the choosing, not by what society deems successful; or something astronomical and my first thought won’t be dying, or cutting or getting drunk or getting high, but to cry & move on.”	1. Emotion regulation (ER) skills and psychoeducation on concepts, connections, decision-making, and diagnose (D_x_) and symptom (S_x_) management 2. Crisis-management including boundary issues and rights and self-speak 3. Support by family, friends, and empathic/understanding professionals 4. Peer support (in PISA group)
Bostik 2007	STBs	SR	No STBs > 6 Mo	Intv. SR	NR	1. Attachment relationships 2. Experiences of attachment 2.1 Acceptance 2.2 Permanent relationship 2.3 Encouragement by others 2.4 Intimacy and closeness 3. Changing self-perceptions
Buelens 2023	SH	1 item, if yes, + 6 FU items	Transition of NSSI-D to non-NSSI	STBs measure	NR	Male Gender (♂) *T*_1_: B = 2·39, SE = 1·16, Wald χ^2^_(1)_ = 4·25, *p* = ·04 *T*_2_: B = 1·60, SE = 0·66, Wald χ^2^_(1)_ = 5·79, *p* = ·02
Buser 2014	SH	SR	Reduction or cessation of NSSI	Intv. SR	NR	1. Recognition of serious physical damage 2. Corrective interpersonal influences 3. Move to healthy surroundings
Claréus 2021	SH	DSHI-9-R[148, 149]	> 5 at *T*_3_	DSHI-9-R[148, 149]	Turning points that impacted previous situation	Recovery turning points: 1. Sense of belongingness 2. Liberation from inhibiting context; 3. Gained perspective 4. Realizing one can deal with adversity 5. Contributing positive life transitions to perceived personal ability
Claréus 2023	SH	DSHI-9-R[148, 149]	No (i.e., 0), infrequent (i.e., 1‒4 instances), or repetitive (i.e., ≥ 5 instances) at *T*_3_	DSHI-9-R[148, 149]	NR	* Continuation of repetitive NSSI associated with fewer positive events (OR = 0·49–0·68, *p* = ·05-·07) and more negative events 1–5 yrs. ago (OR = 1·81–1·93, *p* = ·099-·010), vs. full/partial cessation of repetitive NSSI * Continued repetitive NSSI are less likely to reach an important life goal 1–5 yrs. ago vs. PPs with ceased NSSI (OR = 0·43, *p* < ·05)
Everall 2005	STBs	Interview SR	Overcoming suicidality	SR	No longer wanting to die by suicide	1. Moving to new city 2. Identity formation 3. Sense of connection 4. Improved family relations 5. Sense of agency and control 6. Increased self-worth 7. Taking action and risk 8. Change in outlook
Everall 2006	STBs	SR	No STBs for ≥ 6 mo	NR	NR	1. Social relations with peers, parents and extrafamilial adults 2. ER skills 3. Perspective/recognition of personal control 4. Purposeful and goal-directed action
Fenaughty 2003	SA	SR	NR	NR	NR	1. L/B/G people and issues in MSM may reduce feelings of invisibility, alienation and social isolation for L/B/G youth 2. Parental support in changing schools can help bolster resilience 3. School & peer support 4. L/B/G Support Network Participation (shared experience, find collective solutions, respite from hostile home environments, safe sex info) 5. High self-esteem 6. Coping Mechanisms
Gelinas 2013	SH	DSHI[149]	DSH cessation	Q_s_ on reasons for stopping SH	NR	1. Realization of DSH stupidity = futility 2. Distress over scars and neg. attention 3. Change for interpersonal reasons 4. Receipt of help = support 5. Desire for wellness 6. Development of alternative coping **Strategies that aid recovery:** 1. Positive coping behaviors 2. Seeking professional help 3. Negative coping behaviors 4. Seeking social support 5.Rationalization during self-talk
Goncalves 2023	SH	SIQ-TR-SF^4^	Past SH > 1 yr	SIQ-TR-SF[150]	NR	1. Resilience: PPs with “Past” NSSI higher levels than “Current” NSSI on Personal Competence (U = 507, *p* < ·005), Self-discipline (U = 549, *p* < ·01), Resilience (U = 531, *p* < ·005) 2. PPs past-NSSI more self-compassion than current NSSI (*p* < ·05, M = 2·67, SD = 0·81 vs. M = 2·25, SD = 0·67)
Gulbas 2019	SA	SA ≤ 6 mo. < *T*_0_	NR	Trajectories of well-being, resilience and tenuous growth	NR	1. Trajectories of wellbeing: positive ethnic identity, effective coping strategies, and changes in their school environment 2. Trajectories of resilience: improvement in self-perception, coping skills through therapy, or relationships with family members and peers over time 3. Tenuous growth trajectory: The cultivation of hope and development of coping skills through a supportive network
Hasking 2024	SH	SR	A history of NSSI, but more than cessation (person-centered approach)	Self-injury frequency (past year: 1 = none, 6 = 5 +), perceived recovery (0–100), urge to self-injure (0–100), and urge to avoid self-injury (0–100)	NR	Disclosure to others
Holliday 2015	SA	ED adm. for SA	Going up/climbing up a ladder	NR	NR	1. Connections to others
Keefner 2020	SA	1 item	SA survival	Process post-SA	Process of seeing new ways to escape	1. Connecting and being heard and helped to change perspective 2. Leaving it all behind; seeing new ways to escape, a sense of control 3. Finding a reason to live which involves being part of a family and helping others 4. Someone who listens and validates PPs feelings
Kelada 2018a	SH	1 item NSSI definition.^1^ PPs SR on NSSI (Y/N)	PPs SR recovery from NSSI on scale, with high scores indicate PP_s_ closer to recovery	NSSI Recovery Scale	NR	1. Recovery unrelated to seeking prof. help 2. Poor family function and maladaptive ER were barriers to recovery 3. Adaptive ER moderated the relationship between poor family functioning and recovery
Kelada 2018b	SH	PPs asked about intentional SH without suicide intention (Y/N)	No pre-set definition was given as this was one of the goals of the study	SR	Lost “urge” or need to self-injure and being able to face triggering situations without turning to NSSI	1. Supportive understanding and non-judgmental parental response was key 2. Negative parental responses hamper recovery incl. neg. emotions, e.g., parental anger, disappointment, and sadness (*n* = 9, 33·3%) 3. Ignoring NSSI of PPs was considered unhelpful to recovery (*n* = 8, 29·6%) 4. Subgroup (14·8% youth) noted parents had not attempted to understand NSSI engagement and demanded they stop 5. Young people SR supportive prof. who tried to understand behavior helpful to the recovery process **Non-helpful MH professionals interactions** 1. PPs felt forced to talk and open up 2. PPs felt no trust of prof. & uncomfortable and misunderstood (*n* = 5, 20%) 3. PPs said prof. lack of empathy unhelpful
Knowles et 2022	SH	SR	NR	NR	See recovery factors for definition	1. ↑coping skills 2. Safe environment, ↑acceptance and understanding @home & school 3. ↑Teacher + peer awareness of SH and how to speak about it 4. ↑Enjoyable + social activities 5. ↓SH thoughts and behaviors 6. Sustaining engagement with therapy
Kolar 2012	STBs	SR	History of SH and/or suicidality	SR	NR	1. Social support by someone who cares 2. Development of coping strategies and beliefs that a trajectory away from homelessness or SH is possible 3. Practical support by SIY agencies.^2^
Kwok 2019	SI	C-SIS[151]	SI reduction	C-SIS[151]	NR	Gratitude
Lewis 2019	SH	ISAS[152]	NR	SR open-ended questions	1. > NSSI cessation 2. NSSI cessation 3. A process 4. Developing resilience 5. Lingering NSSI features 6. Evolution in understanding of recovery 7. Recovery as a subjective experience	1. Absence of NSSI thoughts 2. Increased self‐acceptance 3. Psychological recovery (overcoming D_x_) 4. Increased coping skills to replace NSSI behaviors
Meheli 2022	SH	ISAS[152]	Difficult and multifaceted process that involves but is not limited to developing a repertoire of coping mechanisms, fostering self-acceptance, and building resilience and foster Ψ well-being	SR using open-ended items	NR	1. Prof. support, 3 subthemes: D_x_ + referral to therapy. ER strategies. ↑Self-understanding.2. Informal sources incl. friends, family, and romantic partners, 3 subthemes: (a) support system: PPS encouraged and emotionally supported by close contacts ↑recovery. Social support is crucial to start & sustain the recovery process. (b) compassionate space: no longer feeling alone, self-expressing without judgment, and (c) someone to talk to. PPs felt valued and cared for ↑self-confidence. (d) receiving tangible aid: different activities, distracting PPs, or offering company to avoid acting on the urge
Meheli 2023	SH	ISAS[152]	The process of change from engaging in NSSI to the cessation but is not always defined in terms of complete stopping of the behavior	SR	NR	Reasons to stop NSSI: 1. Addiction to NSSI 2. Others’ expectations 3. Desire to change 4. Negative impact on relationships 5. Wanting one’s scars to heal 6. Negative emotional consequence of engagement in NSSI (feeling wrong/bad)
Muehlenkamp 2018	SH	SITBI-NSSI[153]	No SH	“If not engaged in SI past yr., would you consider yourself “recovered” from SI?”	NR	Older age of onset
Norton 2011	SH	SR during treatment	NR	NR	NR	1. Empathy (someone that listens non-judgmentally). 2. Redirecting energy to outward activities (such as art and poetry) and not keeping them inside and cutting.^3^
Ortin 2019	STBs	12-mo. SI + SA using Mood D_x_ Module of DISC-IV[154], DSM-IV version	Remission SI	no SI at FU	N/A	Comorbid mood D_x_ ↑remission (OR = 14·4)
Özen-Dursun 2023	SH	SR	No SH > 1 yr	SR	NR	1. Professional help 2. Self-care 3. Psychoeducation and personal growth 4. Improving social relations 5. Faith and spirituality
Puotiniemi 2004	SH, SA	SR	NR	NR	NR	1. Emotional support was the most important coping resource for girl: via pen friend, mother, sisters, relatives, and PPs therapist 2. Coping strategies were hobbies: drawing, music, reading, swimming and TV 3. Dissociation: PPs escaped from reality into a fantasy world when everyday life was too distressing 4. Therapist and therapy were important to ♀ as they paid attention to PPs
Real 2025	SI	PANSI[155]	Lower levels of suicidality	Lowe levels on PANSI[155]	NR	Between persons-effects: 1. Outness to family (b = -·07, p* p* < ·001) 2. Family acceptance (b = -0·05, p = ·065)
Redmond 2020	SH	N/A	↓SH frequency & severity	N/A	N/A	1. PP owner over life and choices (freedom), 2. PP made active decisions in recovery process (involvement/ownership), 3. Staff responsibility (setting boundaries, distraction), 4. Co-partnership with client (both have responsibilities and should work together), 5. Adaptive coping strategies (personalized to help make other choices than SH),
Rissanen 2013	SH	1 item	SH cessation	1 item	N/A	1. Realizing the uselessness, irrationality, stupidity, unhelpfulness and unattractiveness of self-cutting 2. 11 Personal factors: ↑life situation, neg. consequences of SH ↓maturation, ↑mood, fear of being discovered, neg. emotional sequels of self-cutting, faith, too weak to self-cut, unwillingness to hurt others, flamenco dancing, alternative ways to release bad feelings 3. 9 social factors: having friends and loved ones, social support, others interfere in SH, discussing problems with others, neg. social sequels of self-cutting, a promise to a significant other, the existence of siblings, and pointlessness of SH in relation to others 4. 6 therapy factors: Psychologist, psychiatrist, unspecified therapy, inpatient care, and care from a school nurse 5. The meanings related to the instruments used to cut oneself (‘disgusting knives’) 6. No specific reason for stopping
Shaw 2006	SH	SR	Cessation of SH	SR	Some PPs had a clear desire to stop SH, others expressed little/no desire to stop, or asserted profound ambivalence	1. Elimination of Ψ S_x_ that gave rise to SH 2. Taking control of lives 3. Understanding of triggers 4. Concern for well-being 5. Concrete actions to care for oneself 6. Increased involvement in life pursuits 7. Social support and relational ties 7.1 someone being there 7.2 role models 7.3 connection 7.4 taking mental health serious 8. professional treatment 9. Disclosure
Szlyk 2021	STBs	SR	↓STBs	SR in intv	Newfound resilience	1. Empathic interpersonal relations (P1): Supporting, Non-stigmatizing or accepting environment: students and staff (P2 + P3) > which leads to ↑perspective (P1) 2. Space and time offered by alternative high school (P1 + P2 + P3) 3. Asking for support/help (P2 + P3) 4. Practical support (e.g., applying for colleges) 5. Safety planning (strategies to deal with difficult days) (P2 + P3) 6. Self-empathy (P3)
Tampus-Siena 2023	STBs	SR	No record or admission of STBs and indicators of mental health > 6 mo	NR	NR	**Care-receiver:** 1. Calling-on 1.1 Recognizing the need for help 1.2 Asking for help 2. Allowing 2.1 Opening up to care 2.2 Responding positively to care 3. Reciprocating 3.1 Appreciating and valuing care 3.2 Compensating care through recovery **Caregiver:** 1. Contemplating 1.1 Making sense of the situation 1.2 Recognizing shortcomings 2. Accepting 2.1 Committing to the role of caregiver 2.2 Compensating for what is missing 3. Responding 3.1 Providing care and support 3.2 Resolving family issues and adjusting family dynamics **Both:** 1.Empowering 1.1 Valuing love, fueling hope, holding on to faith 1.2 Strengthening family relations
Teismann 2016	SI	Item 8 BDI[156]	Remission: no SI at FU	Q_s_	NR	Univariate: 1. Symptom distress (OR = 0·47) 2. Self-efficacy (OR = 1·10) 3. Positive mental health (OR = 1·10) 4. Life satisfaction (OR = 2·29) 4. Social support (OR = 2·57) Multivariate: 1. Positive mental health (OR = 1·07) 2. Social support (OR = 1·92)
Tomicic 2021	STBs	SR: Inclusion criterion	"survive" or stop considering suicide	SR int.: “What do you think made you "survive" or stop considering suicide?”	NR	1. Help with hypervigilance 1. 2. Help with internalized sexual stigma 2. 3. Integration of gender identity into treatment / psychological help.^3^
Tong 2022	SA	SI: “During past 12 mo. did you ever seriously think about committing SI?” SA: “How many times did you SA attempt?”	survived SA	Q_s_	NR	Future Well-being
Tordoff 2022	SH, SA	Item 9 on PHQ-9[157]	↓STBs on Q_s_	Q_s_	NR	Access to puberty Blockers (PBs) or Gender-affirming Hormones (GAHs) as TNB youth
Wadman et al. (2017)	SH	SR	‘What might help you to stop SH?’ and ‘What support/services have been (un)helpful?’)	Intv. Q_s_	NR	Practical costs of cutting: you make a mess and it costs time and money to clean it up
Wagner et al. (2000)	SI	SI: K-SADS[158] for Adol., SI in PPs adm. note, CDI[159, 160] item 9	Improvements in SI	Q_s_	NR	1. ↑Pos. Attrib. styles (Child Attrib. Style Q_s_), 2. Decreased hopefulness 3. Self-esteem was not related to ▲SI 4. Factors related to self: self-worth, overall positive self-appraisal and ER skills 5. Factors related to others: people care and suicide would hurt others 6. Receiving treatment
Wang 2023	STBs	SBQ-R[161]	Transition in suicide risk profile	Latent Transition Analysis	NR	* Adolescents in medium risk and high threat at *T*_1_ reported more control and higher odds of moving to low risk (OR = 1·65) * More regulatory emotional self-efficacy is associated with lower risk (OR = 1·45) compared to PPs with the same profile at *T*_2_ * Adolescents with medium risk-high threat at *T*_1_ age associated with OR = 0·65
Wang 2024	SA	Medical records were screened	Surviving SA: being emotionally stable and willing to be interviewed, being conscious and can express their feelings correctly	Assessed using a telephone call between interviewer and PPs	NR	1. New life insights: cherish life, ↑meaning in life 2. Self-reconciliation: ↑self-acceptance, self-openness and self-understanding 3. Personal empowerment: ↑self-reliance, responsibility, ER 4. Life redesigning: faith in future, plan for the future
Whitlock 2015	SH	NSSI-AT[162]	No NSSI > 1 yr	NSSI-AT	NR	**Qualitative results:** 1. Increases in ER skills 2. Growth of self-awareness, 3. Changes in coping skills or tools 4. Connections with others 5. Maturity **Quantitative results:** 6. ↑NSSI LT frequencies stop less likely (OR = ·28) 7. ↓NSSI functions endorsed (OR = ·92), 8. ↓NSSI forms used (OR = ·93), 9. ↓Likelihood of thinking of oneself as self-injurer (OR = 0.54) 10. Acknowledgement of perceiving NSSI as a problem in one’s life (OR = 1·40) 11. Gender♀ (OR = 1·55) 12. Older age (OR = NR) 13. Formal therapy seen as helpful (OR = 1·45) 14. Higher quality peer support (OR = 1·25), 15. Meaning in life (OR = 1·21), 16. ↑Life satisfaction (OR = 1·22), 17. Effective ER strategies (OR = 1·39)
Zeller 2015	STBs	IDAS[163]	↓STBs	↓IDAS	N/A	Self-compassion
Zortea 2019	STBs	Inclusion criteria	No longer being imminently suicidal	N/A	N/A	1. Positive relations helped develop healthier coping strategies and ER skills 2. Positive romantic relations were key to rebuilding self-worth and self-acceptance 3. Reciprocal contribution to friendships emerged as another key protective factor
Davis 2018	**PhD dissertations**					
Gelinas 2015	SA	SR	SA survivorship	SR	NR	1. Want to help others by sharing stories 2. Gratitude for life 3. Accepting lesbian identity 4. Attempt Integrated (as part of their lives) 5. Motherhood **Other factors mentioned:** 1. Sense of connectedness and belonging 2. Knowledge of other choices 3. Speaking out about suicide 4. Afraid of death 5. Lessons left to learn
Roberts 2019	SH	DSHI[149]	No SH ≥ 6 mo, believe they are fully recovered	Recovered Individual Screener Q_s_	A process, which begins long before someone actually ceases their SH behaviors and continues long after cessation	1. Pos. contribute to others or worthwhile cause 2. Emotional support (feeling loved and sensitivity when discussing SH) 3. Physical support 4. Informational message for public: (a) SH is serious (b) SH not just for attention, (c) information on other strategies helpful in dealing with emotions or situations (esp. if info comes from PWLE) 5. Contact with mental health prof **Themes starting the recovery process:** 1. Desiring wellness 2. Admitting SH is not aligned with wellness, but a problem 3. Taking steps towards wellness: taking action **Factors that support recovery:** 1. Adopting a new future-oriented perspective and self-truths helped resist SH urges 2. Speaking openly about SH and accepting support 3. Engaging in positive activities and distractions: coping strategies Long-term strategies incl. preventive activities to improve emotions and well-being In the moment coping strategies involve activities that dissuade/distract from SH urge **Lingering effects after recovery:** 1. “Recovery” meant being stronger than remaining urges to SH 2. PP_s_ must be very deliberate and purposeful about staying emotionally healthy, avoiding triggers, and knowing their own limits 3. Positive effects: improvement in PP_s_ self-knowledge, social supports, and persistent mental wellbeing
	SI	1 item	Stop thinking about suicide	Q_s_	NR	**Why recovery:** 1. Positive life circumstance / event 2. Mental health care facilities 3. Social support 4. Religious / spiritual change **Factors that aided in recovery:** 1. Social support 2. Altruistic responsibility entailed caregiving for a human or animal 3. Personal responsibility involves taking self-directed initiative for oneself, e.g., a job 4. Possessing a sense of protective accountability by sparing family members the pain of losing a loved one to suicide

One study into the role of physiological stress-responses in recovery from suicidality met al.l inclusion criteria, but these results were excluded as they were statistically non-significant [164].

Abbreviations: ♀ Women. ♂ Men. Ψ psychological. ▲ change. *Adm* Admission. *Attrib *attributional, *BDI* beck depression inventory, *C-CIS CN* version of the suicidal ideation scale, *CDI* children’s depression inventory^2^, *Disc* discontinuous, *DISC-IV* diagnostic interview schedule for children version-IV, *DSH* deliberate self-harm, *DSHI* deliberate self-harm inventory, *DSHI-9-R* revised deliberate self-harm inventory.^2,3^ *DSM-IV* diagnostic and statistical manual, IV, *D*_x_ disorder diagnosis, *ED* emergency department, *ER* emotion regulation, *FU* follow-up, *IDAS* inventory of depression and anxiety symptoms, *Inc* including, *Intv* Interview, *ISAS* inventory of statements about self-injury, *K-SADS* kiddie schedule for affective disorders and schizophrenia, *M* mean, *Med* medical, *MH* mental health, *Mo* months, *MSM* Mainstream media, *LGBT* Lesbian, Gay, Bisexual, Transgender, *LT* Lifetime, *N* number of participants, *Neg* Negative, *NR* Not reported, *NSSI* Non-suicidal Self-injury, *NSSI-AT* Non-Suicidal Self-Injury–Assessment Tool, *NSSI-d* discontinued non-suicidal self-injury, *PANSI* Positive and Negative Suicide Ideation, *PHQ-9* Patient Health Questionnaire-9, *PPs* Participants, Prof(s) Professional(s), *PWLE* Person with lived experience, *Q*_*s*_ Questionnaires, *N/A* Not Applicable, *NY* New York city (US), *OR* Odds ratio, *Psychother* psychotherapy, *R* range, *SA* suicide attempt, *SB* suicidal behaviors, *SBQ-R* suicide behaviors questionnaire-revised, *SD* standard deviation, *SH* Self-harm, *SI* Suicide ideation, *SIQ-TR-SF* Self-Injury Questionnaire - Treatment Related - Short Form, *SITBI-NSSI* Self-Injurious Thoughts and Behaviors Inventory-NSSI Module, SR self-report, *SRAS* suicide recovery ability scale, *SS* semi-structured interview, *STB* suicidal thoughts and behaviors, *S*_*x*_ Symptoms, *T*_*0*_ baseline, *SIY* Street-involved Youth, *SRAS* suicidal recovery ability scale, *T*_1_ follow-up, *TNB* transgender and non-binary, *Wks* Weeks, *Y/N* Yes or no, *Y.O* years old, *Yrs* years.

^1^ SH refers to directly and intentionally hurting oneself, such as by cutting, burning, or excessively scratching without the intention of killing oneself. ^2^ Gaining stable housing is crucial support for PPs to make the positive changes PPs discussed. ^3^ Themes discussed in the results are not directly related to recovery from STBs but to treatment in general. Only in discussion these themes are linked to STBs. ^4^ Reduction or cessation of NSSIs without psychotherapy or medical help.

*ISO-codes*: AU=Australia. BE= Belgium. CA= Canada. CN= China. CL= Chile. DE=Germany. FI= Finland. HK= Hong Kong. IL= Israel. IN= India. NZ = New Zealand. PH= Philippines. PR= Puerto Rico. PT= Portugal. SE= Sweden. UK= United Kingdom. US= United States of America.

### Characteristics of included studies

The key study characteristics are summarized in Table 1. The studies were conducted in the United States (k = 17), Canada (k = 9), and in the United Kingdom (k = 3). Participants of the 50 included studies were 7–62 years of age (Mean age = 20.4, reported in k = 41 studies), and were typically 15 years when they experienced suicidality (k = 20). Between studies this recall time ranged from 8 days to over 14 years.

Most studies were qualitative (k = 28) and described semi-structured interviews (k = 17). Over half of the quantitative papers (k = 22) were cross-sectional (k = 11). All aspects of suicidality were covered, although most studies focused on self-harm (k = 25), and only five studies focused explicitly on suicidal ideation. Most studies took the perspective of recovered youth (k = 49), but sometimes also researcher or clinician perspectives were reported (k = 1), or both (k = 1).

### Quality assessment

The study quality assessment (see Supplementary Table S2 and S3.) resulted in an inter-rater percent agreement of 77% (total number of agreements on criteria/(total number of criteria * papers)), which was fairly comparable between studies; for qualitative studies by CASP, it was 80% and quantitative study quality by QUIPS was 75%. One study was excluded based on poor quality. Some notable differences are discussed per instrument. Qualitative study quality (CASP) scores were reasonably on statement of aims, or adequately when their research question was quite broad. The relationship between the participants and researcher were not adequately described in several studies, which made it difficult to rate this CASP criterion. Quantitative study quality appraisals (QUIPS) showed that few studies included confounding variables and study attrition rates (apparently 100% of participants were available for follow-up), and statistical methods to handle missing data were also rarely described.

### Data synthesis (description of results)

Below we discuss recovery from suicidality and implicated factors in four sections. First, we review the definition of recovery as stated by the study participants and by the suicidology researchers. Second, we review the factors that contributed to recovery. Third, we outline which factors are related specifically to long-term recovery. Lastly, we describe barriers to recovery.

### Recovery and its definition

The assessment and the definition of recovery by researchers and participants (k = 13) is provided in Table 1.

#### Definition of recovery by study participants

Participants defined or described recovery from suicidality quite heterogeneously in thirteen studies, and often as a process rather than endpoint (k = 7; [68–70, 80, 86, 92, 94]). People with lived experience of suicidality showed no consensus on whether recovery was a complete cessation of self-harm (remission) or the reduction of urges hereof, such as thoughts and behaviors, which varied even within study samples. Participants typically indicated that for them recovery went beyond reduction or complete cessation of suicidality (k = 8; [68–70, 74, 75, 80, 89, 92, 104]).

Recovery was described as a gradual process that could span multiple years, in which one was “able to do [perform] daily tasks” or was “more comfortable with myself” or was “living a life that [was] really mine by the choosing, not by what society deems successful; or something astronomical and my first thought won’t be dying, or cutting or getting drunk or getting high, it will be to cry and move on” [70]. Others wrote that “recovery has lots of ups and downs” [94], “experiencing a process of gradual transition and growth in their sense of personal agency and self-responsibility” [69], or “recovery isn’t linear; it’s a big squiggle on the page” [89], and “what I couldn’t do before, I can do now” [75], and “it’s kind of a work in progress, you know?” [88]. Others described recovery as a process of seeing new, often more healthy ways to escape rather than resorting to self-harming [86]. Recovery was also seen as an active process rather than a passive one: “My concept of recovery is it depends on the person. You open up yourself to recover. It’s a choice. It’s like spiritual. It’s a choice. It’s responsibility.” [68].

Studies on the meaning of recovery focused predominantly on self-harm (12/13 = 92%). The process of recovery was often difficult to distinguish from contributing factors as participants described recovery as ‘better coping skills’ or ‘a safer environment’ and an ‘increase in enjoyable social activities’ [89], or as ‘newfound resilience’ [92, 104]. These factors were also mentioned as contributing to recovery, as described below.

#### Recovery operationalization by researchers

Recovery was operationalized by most researchers (both in qualitative and quantitative studies) as “no longer engaging” in self-harm or suicidal ideation or the urge to attempt suicide (k = 20), or it was measured as a reduction in self-harm or suicidal ideation (k = 11) or either reduction or complete cessation (k = 3). Some authors (k = 3) mentioned they only included participants who considered themselves to be recovered [87, 92, 94]. Lastly, Hasking & Lewis [84] specifically identified patterns of recovery in a quantitative manner and described recovery as a multidimensional construct beyond the mere cessation of behaviors or urges. Recovery could also include cognitive and emotional recovery (via changes in thoughts, and emotional regulation), or as a shift in identity when individuals no longer defined themselves by their self-harm. Additionally, recovery involves adopting healthier coping strategies and improving overall well-being. Since individuals experience recovery in diverse ways, recovery is best understood as a flexible and evolving process rather than a fixed state.

Our synthesis of how people with lived experience and researchers understand recovery results in the consensus definition of recovery as an idiosyncratic subjective experience and a process that may span many years. Additionally, recovery is not an intrapsychic process only, but also requires adaptations in your environment, which involves other people, places and situations as well.

### Factors related to recovery

**Fig. 2:**
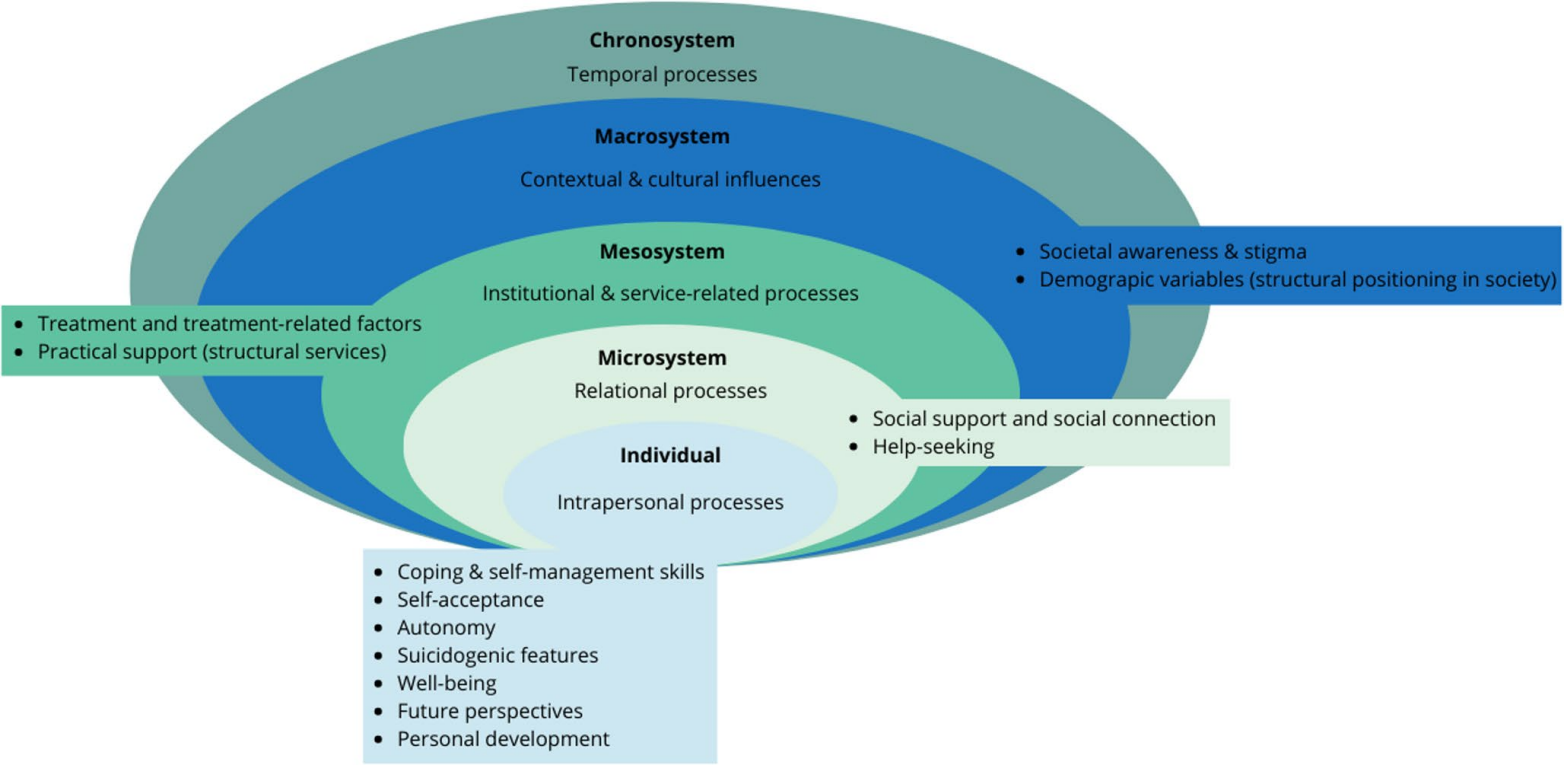
Recovery from youth suicidality: A social–ecological framework of multilevel facilitators. *Note.* This model was adapted from Bronfenbrenner’s socio-ecological model [115]

The 95 factors related to recovery from suicidality among youth we show in Table 2 and were grouped into 13 categories: Social support and connection (k = 32), including Treatment related factors (k = 17), Coping and self-management skills (k = 27), Self-acceptance (k = 18), Personal development (k = 15), Future perspectives (k = 15), Suicidogenic features (k = 12), Autonomy (k = 9), Help-seeking (k = 9), Well-being (k = 8), Practical support (k = 4), Demographic variables (k = 4), and Societal awareness and stigma (k = 4). These thirteen empirically derived recovery categories were mapped onto Bronfenbrenner’s (1979) multisystemic socio–ecological model to illustrate multilevel recovery processes (cf. Ungar et al., 2013; [115]), which (can) involve multiple systems with which an individual interacts. Intrapersonal domains included coping and self-management skills, self-acceptance, autonomy, suicidogenic realizations, and well-being. Relational processes within the microsystem comprised social support and connection and help-seeking from close others. Institutional and service-related processes at the mesosystem included treatment-related and practical support factors. Broader contextual influences at the macrosystem level included demographic positioning and societal awareness and stigma. Together, these interacting layers represent multilevel processes supporting recovery from suicidality (see Fig. 2).

**Table 2:** Recovery factors per study

Categories & subfactors	Explanation	K	Study references
Social support and connectedness			
Informal support	Family and peers	28	[24, 68–71, 73, 75–77, 79–81, 83, 85, 87, 89, 90, 93, 94, 98, 101–105, 109, 112, 114, 116]
Formal support	Mental health professional	24	[64, 68–71, 75, 77, 80, 81, 83, 85, 88–90, 94, 98, 101–104, 109, 112, 116]
Peer support	People with lived experience	6	[70, 79, 94, 104, 110]^1^[80]
Religion and spirituality	Religion and spirituality	8	[71, 76, 86, 90, 101, 102, 105, 116]
*Specific aspects of social support*			
Connection	Feeling connected to other people	10	[71, 75, 77, 78, 80, 83, 85, 86, 103, 112]
Unconditional support	Support is given despite circumstances^2^	2	[71, 80]
Feeling heard/listened to	People listen when I have an issue	4	[77, 81, 86, 96]
Accepting environment	People accept me for who I am	10	[64, 71, 77, 79, 89, 94, 104, 114]^8^[86, 99]
Encouragement	People encourage me to overcome suicidality and develop skills and strengths	2	[71, 80]
Greater awareness	People with more knowledge on suicidality and how to deal with it	6	[79, 80, 89, 94, 104, 105]
Talking/disclosure	People converse and talk with me about suicidality or other issues. Telling other people about suicidality	6	[69, 80, 84, 88, 101, 103]
Social activities (increase)	More activities with others	1	[89]
Family functioning	Properties and transaction patterns that distinguish healthy families	1	[87]
Resolving family issues	Resolving family issues	2	[105, 110]
Stopped by others	Others intervened during SH, SA	1	[101]
School	Supportive or understanding school environment	2	[71, 104]
Means restrictions	Removing objects that could be used for SH or SA	1	[80]
Disapproval by other	Negative sentiment about suicidality	4	[73, 80, 93, 101]
Contributing to lives of others	Helping others	8	[69, 76, 77, 80, 86, 102, 109, 114]
Not wanting to hurt others	Not wanting to hurt, stress, or concern others	10	[69, 73, 81, 93, 101–103, 109, 110, 112]
Factors related to treatment			
Access to treatment	Ability to get referral or access to treatment	2	[83, 93]
Medication	Receiving medication	4	[75, 94, 103, 109]
Assessment	Screening of current level of suicidality	1	[68]
Non-specific element of treatment	Therapist empathy and understanding	4	[70, 96, 98, 103]
Intervention	Having a suicide specific intervention	1	[68]
Symptom reduction & management	Reduction of symptoms other than suicidality	6	[68, 70, 92, 101–103]
Crisis management & Safety Planning	Learning to identify crisis and coping strategies	2	[68, 70]
Psycho-education (for self)	Deriving a greater understanding of suicidality	4	[80, 94, 103, 116]
Service user involvement	Co-partnership between client and therapist	1	[100]
Service user freedom	Client has ownership over life and choices	1	[100]
Staff responsibility	Setting boundaries, distraction	1	[100]
Previous STBs	Lifetime frequency or past SH	1	[112]
Diagnosis	Receiving a diagnosis	3	[70, 90, 94]
Comorbidity	Comorbid mood/disruptive D_x_ next to suicidality	1	[97]
Coping skills and emotion regulation			
Coping skills in general	Coping in general (no specific skills)	16	[69, 80, 81, 83, 86, 87, 89, 90, 92, 96, 100, 101, 103, 112, 114, 116]
Non-adaptive or negative coping	Coping skills that are not adaptive	1	[81]
Distraction	Doing activities when feeling suicidal	9	[77, 80, 81, 94, 96, 98, 101, 104, 116]
Rationalization	Suicidality is unhealthy or availability other options	1	[80, 81]
Attributional style	How one explains causes of events	1	[109]
Skills due to personal ability	Re-imagining events as accomplishments	1	[75]
Emotion regulation	Learning to express and handle emotions	14	[70, 77, 78, 80, 82, 83, 87, 93, 94, 103, 109, 110, 112, 114]
Dissociation	Escaping into own reality in the mind	1	[98]
Self-acceptance			
Self-worth	Feeling worthy of living	5	[71, 78, 102, 109, 114]
Self-acceptance	Accepting yourself as you are.	14	[71, 76, 80, 81]^3^[77, 79, 83]^4^[64, 94, 103, 109, 110, 114]^8^[86]
Self-appraisal	Recognizing positive qualities in oneself	1	[109]
Self-esteem	Valuing yourself	3	[77, 80, 102, 109]
Self-compassion	Kind and comprehensive attitude to self during pain or failure, as part of the larger human experience and awareness.	2	[82, 113]
Self-empathy	Not being over-demanding when feeling unwell	1	[104]
Self-understanding	Gain a better understanding of oneself	1	[77, 94, 110, 112]
Hormonal treatments	Access to puberty blockers & gender-affirming hormones	1	[107]
Personal development over time			
Identity development	Become the person you want to be	1	[78]
Internalizing of behavior	Accepting behavior as part of personal experience, but not as a part of their personality	2	[76, 112]
Maturation	Growing over need to continue suicidality	5	[76, 93, 101, 103, 112]
Wanting to have a family	Wanting to have a family	1	[109]
Parenthood	Being a parent (caring for someone else)	4	[76]^5^[81]^5^[102]^5^[86]
Change in environment	Moving away from current environment	10	[69, 71, 73, 75, 77, 78, 86, 101, 102, 112]
Life events and goals	Both positive and negative life events that occurred or goals that were achieved	3	[74, 75, 102]
Future perspectives			
Hope	Believe in better things to come	5	[71, 80, 83, 109, 110]
Perspective in general	Realizing better things may come	7	[69, 75, 77, 80, 104, 109, 110]
Positive thinking	Thinking of things that are going well	1	[69]
Gratitude	Feeling thankful for life	3	[76, 91, 110]
Future and goal-directed action	Working towards something, such as getting a job, or focusing on things to come rather than current pain	8	[74, 75, 77, 78, 80, 89, 103, 110]
Life satisfaction	Feeling satisfied with life in general	1	[112]
Meaning in life	Participant found more meaning in life	2	[110, 112]
Suicidogenic features			
Addictiveness of behaviors	Perceived dependence on self-harm	2	[80, 112]
Meaninglessness of behavior	Realization that suicidality does not solve problems/issues	3	[76, 101, 103]
Awareness of suicidality problem	Recognition of suicidality problem and need for help	6	[68, 80, 81, 103, 105, 112]
Fear of death	Being afraid to die	1	[76]
Practical costs of SH	Distress over scars, negative attention, blood loss, going to ER	7	[73, 81, 93, 101, 104, 108, 112]
Autonomy			
Agency and control	Experience control over life and choices	8	[69, 71, 75, 77, 78, 103, 110, 111]
Awareness of choice	Awareness of other options than suicidality to handle issues	2	[70, 76]
Help-seeking			
Accepting help	Accepting help	2	[80, 105]
Asking for help	Asking for help	8	[68, 69, 81, 103–105, 108, 110]
Belief support is available	Realization that support is available	1	[71]
Wellbeing			
Wellbeing	a) self-acceptance, (b) life satisfaction, (c) positive mood, (d) infrequent negative mood, and (e) positive relationships with parents.	1	[106]
Positive mental health	The presence of general emotional, psychological and social wellbeing	1	[24]
Resilience	Ability to bounce back from stressors and restore a thriving state	3	[68, 74, 82]
Desire for wellness	Wanting to get better	4	[80, 81, 93, 103]
Practical support			
Practical support	Receiving support with practical aspects	4	[77, 80, 90, 104]
Housing	Receiving support in getting housing	1	[90]
Demographic variables			
Gender	Gender	2	[112]♀ [72]♂
Older age of onset	Being older when suicidality started	2	[95, 112]
Age	Age at time of study	2	[111, 112]
Societal awareness and stigma			
Stigma reduction	Positive portrayal in media, informational messages for the public, reductions in self-stigma	3	[79]^6^ [80]^7^ [64]^8^

We included factors that contributed (positively or negatively) to self-reported recovery of people with lived experience of suicidality. We did not include reasons for recovery, factors that initiate recovery, or factors hypothesized to be related to recovery.

*D*_*x*_ disorder, *ER* emergency room, *FAD* family assessment device (60-items), *K* number of studies, *LGBT* Lesbian, gay, bisexual, and transgender, *SA* Suicide Attempt, *SH* Self-harm. ^1^ LGBT+ peer support. ^2^ This does not mean condoning the behavior. ^3^ LGBT identity. ^4^ Ethnic identity. ^5^ Motherhood specifically. ^6^ Positive media portrayal. ^7^ Informational messages for the public. ^8^ Self-stigma.

### Intrapersonal domains

#### Coping and self-management skills (k = 28)

Acquiring adaptive coping skills was often mentioned (k = 27; [69, 70, 75, 77, 78, 80–83, 86–90, 92–94, 96, 98, 100, 101, 103, 104, 109–112, 114, 116]) as key to recovery, and 22 studies reported specific coping skills: *rationalization* that suicidality is not healthy and that other options are available, *distraction* or performing other activities to distract oneself from thoughts of self-harm or attempting suicide, and adopting an *adaptive attributional style*, e.g. attributing positive events to internal, stable, and global causes [109] and attributing their skills (learned competencies) to personal abilities (or potential or innate capacities; [75]).

Additionally, *emotion regulation* was found to promote recovery (k = 14). Specifically, understanding the role of emotions [70, 80], being able to identify emotions one is experiencing [70], to express these emotions [77, 78, 83, 114], or to handle and tolerate emotions [70, 77, 78, 87, 93, 94, 109–111]. On the other hand, Goncalves and colleagues [82] could not find any differences in emotion regulation strategies between the participants with past and current self-harm.

Acquiring coping skills and emotion regulation was often part of treatment [70, 78, 81, 83, 86, 88, 90, 94, 96, 100], but family, peers and school staff were also helpful for acquiring these skills [70, 77, 80, 81, 94, 104, 105, 114]. Two studies found that some coping skills mentioned by participants and generally considered to be “*maladaptive*” such as substance use (ingesting cannabis when urges became too strong, pulling my hair out, taking drugs) and dissociation, actually helped these participants to recover from suicidality [81, 98].

#### Self-acceptance (k = 21)

Recovery from suicidality associated with self-acceptance and *feeling comfortable* in one’s skin (k = 18; [64, 71, 76, 77, 79–83, 86, 94, 102–104, 109, 110, 112–114], which researchers alternatively described in terms of self-worth, self-esteem, self-compassion, self-appraisal, self-empathy, and self-understanding.

In several studies participants reported the importance of learning to *accept themselves* (as they are), their identities, and their faults. One participant described “I got to a point where I was like, I have nothing to be sorry for, that’s when things really turned around” [103], while another said : The most important thing is that I can accept my shortcomings and see myself as an ordinary person” [110]. Participants described they realized truths about themselves and accepted that they are more than what they thought they were (e.g. being stronger than they are; [81]), could be who they want to be [77], more deserving of wellness [81], not at fault for bad or negative outcomes [109, 110], and recognized positive qualities in themselves [109]. A participant described how it was important to discontinue with negative patterns of guilt and self-blame and to be more self-compassionate on days she felt low [104], while other participants noted that increases in their self-esteem resulted in confidence that they could handle difficult situations [77, 80, 102]. Two quantitative studies showed that self-compassion predicted reductions in suicidality and that youth with past self-harm reported more self-compassion than people with current self-harm [82, 113]. Evidently self-understanding and self-awareness contributed to recovery and fostered a positive outlook on the future and self-identity and sense that they could be who they wanted to be [77, 94, 112]. Additionally, feeling worthy to live and to be who one is fostered recovery from suicidality [109].

Self-acceptance often refers to identity, such as accepting LGBT identity, which proved crucial for recovery from suicidality [64, 76, 80, 81]. Access to Puberty blockers (PBs) and gender-affirming hormones (GAHs, i.e., strategies to enhance gender identity realization) was found to be associated with lower odds of suicidality (OR = 0·27) among transgender and non-binary youths in an urban multidisciplinary gender clinic [107]. The role of identity in recovery was also mentioned by non-LGTB youth as a sense of connection made youth feel confident and assured in their personal identity [71]. Evidently, recovery factors interact, and specifically social support and connectedness prove vital. Similarly, ethnic minority participants described how they had to develop positive connections to their heritage culture and ethnic identity to recover from suicidality [83].

Besides social support, connection and identity being vital for the development of self-acceptance [71, 102, 104, 114], other factors that contributed to self-acceptance were positive experiences and overcoming difficulties in life [77, 80, 102], and treatment and the therapist [94].

#### Future perspectives (k = 17)

Fifteen studies identified perspectives for the future as helpful to recovery from suicidality, such as expectations that their futures will be better (k = 3; [69, 80, 109]), a sense of hope (k *=* 5; [71, 80, 83, 109, 110]), and finding meaning in life (k = 2; [109, 110]). It helped respondents if they were less rigid in their thinking of the future [110]. Eight studies found that actively working on development of perspective by *future and goal-directed action* were considered helpful for recovery from suicidality by participants, e.g., by getting a job, or focusing on things to come rather than current pain [74, 75, 77, 78, 80, 89, 103, 110]. It was unclear whether this skill was something participants acquired themselves, by help of others or in treatment. Additionally, thinking of things that are currently going well was considered helpful for recovery [69], feeling grateful for life [76, 91, 110], and life satisfaction were also helpful for recovery [112].

#### Personal development (k = 17)

Often processes related to personal development (k = 15; [69, 71, 73–78, 81, 86, 93, 101–103, 109, 112, 117]) stimulated recovery such as maturation and growing out of suicidality over adolescence [76, 93, 101, 103, 112], and *personal identity development* and self-actualization [78], which reduced thoughts about suicidality. Also, cognitive skills development contributed to recovery from suicidality. Accepting the suicidal behavior as part of their personal experiences, but not as part of their personality, also contributed to recovery in two studies [76, 112].

Several life experiences or personal development goals during young adulthood associated with recovery, namely parenthood [76, 81, 86, 102], wanting to have a family [109], and a change in environment [69, 71, 73, 75, 77, 78, 86, 101, 102, 112], which allowed youth to separate themselves from a negative situation or context [69, 71, 73, 75, 77, 78]. Sometimes this meant specifically moving away [77], or a removal of negative relationships [112], or moving in with someone who offered them affection or a loving relationship [86, 102], while for others it was simply described as an improvement of the life situation [101], or change in life circumstances [112]. These factors are often also related to a sense of social support and connectedness.

Both adverse events and positive life events could contribute to recovery. Adverse life events were helpful in the sense that they helped participants realize they had the ability to overcome challenges [75], while positive life events enabled the young persons to make changes and different choices [75, 102]. One quantitative study also found that people who were not recovered (continued self-harm) had perceived more negative events, fewer positive events and were less likely to have achieved important life goals [74].

#### Suicidogenic features (k = 12)

Several suicidality-specific or suicidogenic factors fostered recovery [73, 76, 80, 81, 93, 94, 101, 103, 104, 108, 112, 116]. Key examples were distress or negative consequences of suicidal behaviors, one’s realization that suicidal behaviors were futile, and fear of death and prior negative experiences with self-harming, such as worries over scars [73, 81, 93, 101]. When the *emotional gains of self-harming no longer outweighed the negative consequences*, youth might choose a path towards recovery, or a different (less messy) self-harm method [108]. Physical consequences of self-harming behaviors next to scarring [112] were blood loss and messiness [104, 108], pain [101], and infection risk [73]. Often several factors were interrelated, such as a fear of being discovered [101], receiving negative attention when discovered [81, 108], and the possible need for medical attention [73].

Several studies found that realizations about suicidality were important for recovery. Self-harm might have started as a coping mechanism, e.g., to manage stress or reduce negative affect, but participants had to realize that self-harm or suicidal behavior would not solve their issues, and made them feel worse over time [76, 80, 81, 101, 103]. Hence, weighing up the emotional gains against the negative consequences of the suicidal behavior [76, 101, 103] might speak to psycho-education in suicidality treatment as mentioned in four studies [80, 94, 103, 116], which also zooms into suicidality-perpetuating processes. Youth mentioned that being scared of death was a reason to start the process of recovery [76]. More frequent self-harm behavior (lifetime) associated with lower likelihood of recovery [112].

#### Autonomy (k = 10)

Feeling a sense of personal autonomy, agency and control contributed to recovery (k = 9; [69–71, 75–78, 103, 110, 111], and was fostered by enhanced abilities to express and regulate emotions [77]. Youth who experienced control over their life decisions and their response to adverse or stressful circumstances were more likely to recover [69, 71, 77, 78, 103, 111]. Young people who recognized a need for self-reliance tended to recover more [110]. Additionally, youth who actively identified alternatives to suicidality to deal with their issues were more likely to recover [70, 76].

#### Wellbeing (k = 8)

Participants reported that an increased sense of wellness/wellbeing or desire thereof helped them to recover from suicidality [81, 106], in keeping with want to get better and healthy or a desire for change as the first step to recovery [80, 81, 93, 103]. A future sense of wellbeing was achieved by one in seven SA survivors, who said they experienced a) self-acceptance, (b) life satisfaction, (c) positive mood, (d) infrequent negative mood, and (e) positive relationships with parents [106]. Wellbeing just after the suicide attempt predicted future wellbeing years later (OR = 1·22; [106]). Positive mental health, described as emotional, social and psychological wellbeing was also associated with higher remission of SI (OR = 1·07; [24]).

Resilience can be described as an important component of wellbeing, and conceptualized as being able to bounce back from stressors and restore a thriving state [118]. The dynamic process of positive adaptation within the context of significant adversity is called resilience [33, 118], while recovery refers to a stable long-term reduction or absence of symptoms accompanied by functional improvement and well-being [119, 120]. Thus, building resilience facilitates the recovery process [68], and resilience was lower among self-harming youth compared to recovered youth [74, 82].

### Microsystem: relational processes

#### Social support and connectedness (k = 32)

Social support and connection are vital, particularly for people who struggle with suicidality. Most studies (k = 32) reported recovery was fostered by social support and a sense of connection, especially informal support (k = 31) by family, friends/peers, formal support (k = 24) by a mental health professional (e.g. therapists, psychologists, psychiatrists), and support by peers with lived experience (k = 2) or in the religious/spiritual context (k = 8). The specific components of social support and connectedness are explained below.

Every study that investigated interpersonal factors (k *=* 14) found social support helpful for recovery. The six studies that did *not* support the role of interpersonal factors in recovery did not measure them (e.g. they were quantitative studies that tested hypotheses on other factors). Additionally, two studies examined recovery factors during ongoing psychological treatment, and focused on factors that supported their treatment aims [64, 100]. A premise of these studies is therefore that formal support already helps, but that certain elements of treatment might improve these positive effects of formal support.

#### Informal support (k = 30)

Informal support by family, friends and peers is key to recovery from suicidality (k = 31); specifically a sense of connection [71, 75, 77, 78, 80, 83, 85, 86, 103, 112, 117], receiving unconditional support [71, 80], and an accepting environment [64, 71, 77, 79, 86, 89, 94, 99, 104, 114]. Being able to express and discuss feelings and issues is important due to feeling heard (people that listen) and receiving encouragement. Family environment seems particularly important as family functioning and resolving family issues were specifically mentioned [88, 105, 110]. Psycho-education of family and friends also seems a promising strategy as greater awareness about suicidality by others was helpful for recovery (k = 6; [79, 80, 89, 104, 105]) and for restricting participants during self-harming behaviors or plans (k = 2; [80, 101]). Social support was associated with lower odds (OR = 1·92; [24]) of SI.

The desire to not hurt important others by self-harming or attempting suicide (k = 10; [69, 73, 81, 93, 101–103, 109, 110, 112]) was also a factor in recovery, namely, as a reason for deciding against suicide or suicidal behaviors [102, 109]. In addition, three studies found that people who expressed themselves negatively about suicidality to the person struggling with suicidality could contribute to recovery (friends; [73, 80]; unspecified; [93]). This might appear stigmatizing, but helped suicidal youth to realize they did not want to hurt others, which contributed to the recovery process.

#### Peer support from young people with lived experience with suicidality (k = 6)

Youth said peers with lived experience contributed to their recovery (k = 6). Some youth noted that only people who had a similar experience could really understand them [70, 80, 94], and one participant noted that people with similar experiences were more respectful and sensitive towards each other as they came from the ‘same bunch’ [104]. Young gay men said that contact with other LGB youth in general already fostered their recovery from suicidality [79]. Young people also indicated that witnessing the determination of others facing similar challenges can be a powerful motivator, inspiring individuals to persevere in their own journey toward recovery [110].

#### Help-seeking (k = 10)

Asking for help from family and friends or mental health professionals [68, 69, 81, 103–105, 108, 110] and accepting help [80, 105] were important for recovery. Some youth said that others couldn’t be expected to help unless they themselves clearly asked for support [69], and shared that they were feeling suicidal [103, 108]. Professionals often noted that recovery typically began only when youth acknowledged their need for help [68]. Importantly, both informal and formal help needed to be accepted, not resisted, for recovery to progress [71, 80, 105].

### Mesosystem: institutional and service-related processes

#### Formal support (k = 24)

Youth reported that *support* by a mental health professional had promoted their recovery (k = 24; [64, 68–71, 75, 77, 80, 81, 83, 85, 87–90, 94, 98, 101–104, 109, 112, 116]), but not seeking professional help on its own [87]. Often psychotherapy from counselors or psychologists played an important role in youth recovery from suicidality [75, 77, 81]. Being able to talk to a non-judgmental person [64, 88], having someone that cared [90], or paid attention [98] was helpful to them. Several participants found it easier to talk to counselors than family, noting the emotional neutrality of professionals [69].

#### Factors related to treatment (k = 17)

Several studies identified aspects of treatment at (mental) health care services that contributed to recovery (k = 16; [70, 75, 80, 83, 90, 92, 94, 96, 97, 100–102, 109, 116]), next to access to treatment (k = 2; [83, 93]). Specific positive aspects of treatment that were mentioned were symptom reduction and management (k = 6; [68, 70, 92, 101–103]), crisis management and safety planning (k = 2; [68, 70]), suicidality psycho-education (k = 4; [80, 94, 103, 116]), assessment/screening (k = 1; [68]), receiving a suicide-specific intervention (k = 1; [68]), receiving/taking medication (k = 4; [75, 94, 103, 109]), receiving a diagnosis and referral to therapy (k = 1; [94]), and remission from a comorbid mood disorder (OR = 14·4, k = 1; [97]).

For LGB youth the inclusion of their LGB identity in treatment proved important for recovery from suicidality [64]. It proved important to become aware of and alleviate hypervigilance i.e. negative expectations or having a guard up about one’s sexual or gender identity, and to help reduce internalized sexual or gender related stigma [64].

Mental health professionals suggested that service user involvement (co-partnership) and freedom, and setting boundaries were important aspects of treatment of adolescents girls with a high-risk for self-harm and suicidality (e.g. certain risks are accepted but measures for safety must be clearly outlined per service user, and adapted to their situation [100].

#### Common/non-specific aspects of treatment (k = 4)

Several non-specific aspects of treatment also promoted or hindered recovery from suicidality, which are factors that can be found across therapists and interventions or treatments that are not key to the therapy itself [121]. Professionals with an *empathic understanding* were considered important to recovery [70, 96, 103], or simply someone who paid attention to them [98]. A young girl was able to ‘truly express how she felt’ when talking to a professional, due to their empathy [96].

#### Practical support (k = 4)

Practical support during periods with mental health difficulties promoted recovery, including support with housing, planning (i.e. how to order tasks and daily activities), and extra support at school. Recovery was supported by small gestures such as setting up counseling appointments [77, 80], preparing meals [77], financing treatment [80], getting subsidized housing [90], or administrative support in applying for colleges [104]. Such help allowed suicidal youth to worry less about daily hassles and to focus on recovery.

### Macrosystem: Contextual and cultural processes

#### Societal awareness and stigma (k = 3)

Society or the broader public might also play a role in recovery from suicidality as more awareness of suicidality and mental health issues and public stigma reduction via information and positive media portrayals were considered to be helpful [64, 79, 80]. Public attention for suicidality may reduce feelings of invisibility or social isolation, increase acceptance of people with mental illness, and increase help-seeking as both young people themselves and their social environment are more likely to recognize and willing to act on their need for help, such as peers, parents, and teachers, among others.

#### Demographic variables (k = 4)

Results regarding gender were inconclusive, as some studies report men were more likely to recover [72], while others reported that women were more likely to recover from self-harming [112]. Older youth were less likely to recover (OR = 0·65; [111]), while the people with past self-harm were slightly older than people with current self-harm [112]. Additionally, people who started self-harming behaviors at an older age were less likely to have harmed themselves in the past year [95, 112].

### Barriers to recovery

Eight studies [68, 80, 88, 90, 92, 94, 103, 108] described factors that participants mentioned that hampers their recovery, often related to other people [94], which underscores the importance of social support and connection in suicidality. Examples were not wanting to involve or burden others with requests for help [68, 94], as participants wanted to solve their issues themselves [94], or lacked trust [80], or were ashamed about their suicidality [94], or feared stigmatization [68, 94, 103] or previously negative experiences with help-seeking [68, 88, 94], such as unhelpful reactions from parents or friends, and family problems [68] and negative demeanor by doctor/nurses at ER [80, 88, 108]. Other aspects of mental health professionals or treatment that were unhelpful for recovery from suicidality included feeling forced to talk and open up, not trusting the professional, low empathy responses, and feeling uncomfortable or misunderstood [88].

Another important aspect for self-harm specifically was that it provides relief from negative emotions [108] and distress [88], and is perceived by participants as an effective emotion regulation strategy, at least for a period in their lives [88]. Two studies drew parallels between self-harm and addiction insofar that participants experienced self-harm dependence [80, 112], which some described as a hindrance to recovery with many participants experiencing relapse [80], whereas others observed no difference in dependency between self-harming youth versus recovered peers [112]. Nonetheless, the addiction parallel applies to self-harming participants who report stress reduction [88] habituation (i.e. self-harm as automatic distress response) [88, 108], and unsuccessful previous attempts [88] as barriers to recovery. Participants also mentioned how self-harm would always be in the back of their mind as a potential way of dealing with negative emotions [92]. Moreover, when distress became overwhelming their newly-learned adaptive coping mechanisms were often overthrown and participants re-attempted suicide or self-harmed [90].

Other factors mentioned as setbacks to recovery were self-harm as punishment for causing negative emotions in themselves and others [108] and external triggers of self-harm such as depictions of self-harm on social media, seeing scars on others, seeing objects related to self-harm such as knives, or others talking about self-harm [80]. Internal triggers of self-harm included academic stress, and trauma that resurfaced [68].

## Discussion

We reviewed 50 studies of facilitators that foster recovery from suicidality, which describe a multifaceted and non-linear process, shaped by a *dynamic interplay* of personal, social, relational, and contextual facilitators. Recovery was consistently described by youth not as a discrete endpoint or “cure” but as an ongoing, dynamic journey marked by fluctuations, setbacks, and growth, aligning with more humanistic and person-centered approaches that foreground empathy, hope, and personal meaning [122, 123]. While most research operationalized recovery as the reduction or absence of suicidality, lived-experience accounts by young people articulated a broader and more holistic definition, encompassing emotional regulation skills, a sense of belonging, autonomy, identity development, and having a valued place in society [88, 92]. Importantly, participants often highlighted the lingering nature of suicidality and self-harm urges, even after sustained periods of non-crisis. Several studies noted that youth anticipated remaining in an ongoing “maintenance” or relapse-prevention phase for the foreseeable future [124]. This has led some researchers to compare aspects of self-harm to addictive behavior patterns, emphasizing the need for long-term, supportive care [125]. Consequently, recovery efforts must include strategies for managing vulnerability over time rather than presuming complete risk elimination. These converging findings provide a basis for reconsidering how recovery from suicidality is conceptualized within suicidology. Accordingly, we now discuss how current theoretical and clinical frameworks may be expanded to better reflect the empirically observed features of youth recovery.

### Theoretical context and multilevel interpretation of recovery processes

Overall, the recovery processes identified in this review align closely with contemporary theoretical models of suicidality and recovery. Across studies, recovery was not driven by a single factor but by interrelated interpersonal, cognitive, emotional, and identity-based processes. This constellation mirrors the COURAGE framework’s proposition that recovery involves non-linear movement across connectedness, acceptance, meaning-making, empowerment, and identity optimization, alongside established theories of suicidal behavior that emphasize belonging, burdensomeness, psychological pain, and entrapment [46, 47]. This convergence provides a theoretical basis for interpreting recovery as a multilevel process embedded within individuals’ social ecologies. Accordingly, we organize the core recovery domains in line with Bronfenbrenner’s socio–ecological framework, reflecting interacting intrapersonal, relational, institutional, and societal influences (see also Fig. 2).

### Intrapersonal level: coping, emotion regulation, autonomy, identity and meaning

At the intrapersonal level, recovery involved strengthening internal coping skills and emotional regulation strategies that enable youth to navigate distressing thoughts and feelings [45]. These capacities were closely intertwined with growing self-acceptance, autonomy, identity development, and future-oriented meaning-making [78, 80, 84, 92]. Such processes align with theoretical models describing suicidality as arising from unbearable psychological pain, aversive self-perception, and entrapment. Shneidman’s psychache model [48] and Baumeister’s escape theory [49] emphasize the role of self-directed distress and perceived self-defectiveness in suicidal behavior, while Williams’ cry-of-pain model [50] highlights defeat, entrapment, and absence of perceived rescue. Recovery processes that involved self-compassion, identity acceptance (including sexual and gender identity), and recognition of self-worth appear to transform these internal appraisals, reconstruct livable self-narratives, and restore perceived alternatives to suicide. In COURAGE terms, these changes reflect optimizing identity, understanding oneself, rediscovering meaning, and empowerment [46]. Consistent with this, participants described recovery as requiring active personal engagement rather than passive symptom reduction, underscoring the importance of autonomy and agency in sustaining well-being and relapse prevention [124–126].

Interestingly, suicidogenic features themselves sometimes contributed to recovery, such as distress about the consequences of self-harm, fear of death, or recognition that suicidal behavior did not resolve underlying problems. These experiences can be understood as cognitive-emotional reappraisals that shift decisional balance away from suicide and weaken the perceived efficacy of self-harm as an emotion-regulation strategy, corresponding to the COURAGE process of “choosing life.”

### Relational level: social support and connectedness

At the relational (microsystem) level, social support and connectedness emerged as the most coherently reported recovery facilitators, consistent with previous reviews [43, 45, 46] and theoretical models like the IPTS [47]. Informal support from family and peers, peer support from individuals with lived experience, and empathic professional relationships offered emotional validation, hope, and belonging, directly counteracting social disconnection and perceived isolation. Experiences of being heard, accepted, and validated map onto COURAGE processes of acceptance and growing connectedness. Despite adolescents’ developmental shift toward peer importance or reliance, the family environment remained critical in recovery, underscoring the importance of family-inclusive prevention and intervention strategies. In addition, despite adolescents’ developmental shift toward peer reliance, the family environment remains critical in recovery, underscoring the need for family-inclusive prevention and intervention strategies [127]. At the same time, peer relationships - while predominantly experienced as positive - also carried potential risks, including contagion of suicidal thoughts or behaviors, highlighting the context-dependent nature of recovery influences and challenging simplistic risk–protection dichotomies. Social connectedness was further intertwined with developmental tasks of identity formation and autonomy, reinforcing the importance of embedding recovery supports within youths’ broader social ecologies [128]. Barriers at this level further reinforced theoretical linkages: shame, fear of burdening others, mistrust, and negative help-seeking experiences reflect persistent perceived burdensomeness and thwarted belongingness, while continued reliance on self-harm for emotion regulation and relapse under overwhelming distress reflect ongoing entrapment and habitual coping loops.

### Institutional and service-related level: treatment and practical support

At the mesosystem level, treatment-related and practical support factors played an important role in recovery. Help-seeking and acceptance of professional support often marked turning points, consistent with COURAGE’s empowerment process and dynamic models emphasizing shifts in perceived control. However, our findings also showed that fostering autonomy and self-acceptance was often neglected in treatment approaches focused narrowly on symptom reduction [126]. While lived-experience accounts as retrieved in studies’ empirical results sections formed the core of this synthesis, one included study also examined mental health professionals’ perspectives on recovery. Clinicians tended to emphasize symptom stabilization, risk management, and service engagement, whereas young people highlighted autonomy, identity development, social belonging, and meaning-making as central to their recovery. This divergence underscores the importance of integrating youth-defined recovery goals into clinical practice and research, and highlights the potential for misalignment between professional and lived-experience conceptualizations of recovery. Attuning to these differing perspectives may improve therapeutic alliance and enhance engagement in recovery-oriented care.

### Societal and structural level: stigma, awareness and cultural context

At the macrosystem level, societal stigma, public awareness, and access to practical resources shaped recovery contexts. Consistent with findings from Milner et al. [129] and Stack [130], societal-level interventions such as reducing stigma, improving social welfare, and addressing economic inequalities have the potential to enhance recovery and reduce suicide mortality. While public attitudes toward suicide can influence recovery processes, it is important to distinguish between disapproval of suicidal behavior and stigma associated with suicide, which is linked to reduced disclosure, lower rates of help-seeking, and poorer recovery outcomes [131]. Promoting public disapproval without careful consideration risks reinforcing stigma and inadvertently hindering recovery. This complex relationship underscores the need for anti-stigma efforts that encourage open dialogue, reduce judgment, and support individuals in seeking help.

#### Synthesis

Taken together, recovery from youth suicidality appears to involve dynamic movement across connectedness, meaning-making, identity reconstruction, emotion regulation, and agency, unfolding within interacting intrapersonal, relational, institutional, and societal systems. The close mapping between empirically derived recovery factors and contemporary suicide and recovery theories strengthens confidence in the interpretability of the present findings and highlights multilevel leverage points for intervention development.

### Developmental context

The recovery processes identified in this review are also shaped by the developmental context of adolescence and emerging adulthood. These life stages are characterized by identity formation, autonomy-seeking, exploration of social roles, heightened sensitivity to peer evaluation, and ongoing maturation of emotion-regulation capacities [128]. Many of the dominant recovery factors, including social belonging, identity acceptance, autonomy, and future-oriented meaning-making, map directly onto these normative developmental tasks in adolescence and emerging adulthood. At the same time, risk-taking tendencies, heightened emotional reactivity, and still-developing regulatory control may amplify vulnerability to suicidal crises while also shaping how young people engage with coping strategies and support systems. This developmental framing helps explain why peer relationships, family dynamics, and identity-related processes emerged so prominently across studies. It also suggests that some recovery mechanisms observed here, such as meaning-making, self-acceptance, and emotion regulation, may represent general processes of psychological recovery that extend beyond youth, while their specific expressions are developmentally situated.

### Strengths, limitations, and suggestions for future research

This review offers a rare, wide-angle perspective on youth recovery from suicidality, by purposefully combining quantitative and qualitative evidence published in any language and applying rigorous quality appraisal tools (CASP for qualitative work, QUIPS for quantitative studies), alongside researcher triangulation at every stage of screening, extraction, and synthesis. This wide study range ensured that even when primary papers examined recovery within broader mental-health contexts (rather than suicidality alone), each of the thirteen recovery-related factor categories remained represented, attesting to the robustness and generalizability of the synthesis.

Notwithstanding these strengths, several limitations characterize this review. Included studies relied on retrospective and cross-sectional designs, vulnerable to recall bias, socially desirable responding, and framing effects, precluding inferences about causal pathways or within-person change over time. Furthermore, several included studies relied on retrospective adult accounts of earlier suicidal experiences, which may reflect reconstructions shaped by later developmental stages and accompanying new insights. Longitudinal research following individuals across key life transitions is needed to clarify how recovery processes evolve over time and which early recovery experiences confer enduring protection against later suicidality. Recovery itself is nonlinear, dynamic, and marked by recurrent self-harm and fluctuating suicidal thoughts [92], yet very little longitudinal, youth-led, mixed-methods work exists to chart trajectories, turning points, and mechanisms of sustained improvement. Moreover, designs often depend on self-reported perceptions of what helped or hindered recovery. Subjective narratives, in the absence of objective markers, complicate interpretation of facilitators of recovery. Further blurring interpretation is the frequent conflation of recovery conditions (e.g., social support, therapeutic alliance) with outcomes (e.g., cessation of self-harm, functional gains) [34].

Another constraint is the lack of diversity in study populations. We were unable to examine gender differences in recovery processes; most research was restricted to WEIRD samples - those who are Western, Educated, Industrialized, Rich, and Democratic [132], with few studies addressing sexual orientation, gender identity, race/ethnicity, or socioeconomic aspects. This limits generalizability and underscores the need for culturally sensitive research. Research conducted in Belgium and the Netherlands suggests that in regions with lower suicide rates, self-stigma and shame were lower, and informal help-seeking intentions higher, indicating societal norms and public attitudes can be protective [133, 134].

Current research tends to overlook the highly dynamic nature of suicidality. Both suicidal ideation and self-harm are known to fluctuate significantly over time [135, 136], are highly recurrent [137–141]. Recovery must therefore be studied longitudinally, yet most studies remain single-snapshot [44], with only two studies in this review mentioned factors that contribute to long-term recovery, and even these studies were cross-sectional. Longitudinal and mixed-methods designs are urgently needed to assess within-person change, model recovery trajectories, and examine the interplay of protective and risk factors over time [142, 143].

Also, the precise influence of developmental context of recovery warrants attention. Adolescence and emerging adulthood are periods marked by rapid identity formation, autonomy-seeking, and psychosocial vulnerability [128]. Recovery processes identified in youth may represent early stages in longer trajectories rather than permanent resolution of vulnerability. Developmentally attuned frameworks are necessary to contextualize recovery within the tasks and transitions of each life stage, improving ecological validity and ensuring lived experiences are integrated into research design and interpretation [144]. At the same time, vulnerability to suicidality may persist or re-emerge later in life. Recovery processes identified in youth may thus represent early phases within longer and non-linear recovery trajectories rather than permanent resolution of risk. Furthermore, several included studies relied on retrospective adult accounts of earlier suicidal experiences, which may reflect reconstructions shaped by later developmental stages. Longitudinal research following individuals across key life transitions is needed to clarify how recovery processes evolve over time and which early recovery experiences confer enduring protection against later suicidality.

Future studies should prioritize longitudinal, mixed-method designs that distinguish recovery processes from outcomes, disaggregate intervention components, and incorporate youth-led perspectives. Such approaches would offer richer, more accurate insights into how diverse young people navigate recovery, and how systems (clinical, familial, societal) can better support them. Although studies primarily focused on recovery experiences rather than treatment efficacy, participants frequently described therapy, medication, or service encounters as part of their recovery narratives. Excluding treatment-focused intervention studies may therefore limit conclusions regarding specific therapeutic mechanisms, which should be addressed in future targeted reviews [145].

Importantly, the facilitators of recovery identified rarely occurred in isolation. For example, peer relationships with others who had lived experience often supported identity reconstruction, while successful help-seeking experiences facilitated renewed trust in social connection. Future research would benefit from examining how such processes interact dynamically over time rather than treating recovery elements as independent factors.

## Conclusion

Recovery is an idiosyncratic and partially subjective experience and a process that can unfold over many years. Social connections including support from family and friends, peers with lived experience with suicidality, and professional support in treatment, consistently emerged as central. While our categorization offers a detailed understanding of these factors, their effects are interrelated, underscoring the importance of multidirectional influences. Recovery is not solely an individual journey, but one shaped by broader social and cultural contexts. Given the central role of interpersonal dynamics, a *social-ecological perspective* on youth suicidality is essential. Accordingly, treatment and recovery efforts must extend beyond the individual, embracing developmentally informed and culturally inclusive approaches that actively engage youth as partners in care.

## Supplementary Information

Below is the link to the electronic supplementary material.

Supplementary Material 1

## Data Availability

No datasets were generated or analysed during the current study.
