# Supplementary material for: Recovery from suicidality in youth aged 6 to 25: a review of 50 quantitative and qualitative studies
Source: Eur Child Adolesc Psychiatry. 2026 Apr 11;35(6):1753–88. doi: 10.1007/s00787-026-02991-y (PMC13337683; doi:10.1007/s00787-026-02991-y)
Supplement: Supplementary file 1 — Supplementary Material 1 [file 787_2026_2991_MOESM1_ESM.docx]

**Supplemental Materials.**

**Table S1. Search Strings per database.**

| **PsycInfo** |  |
| --- | --- |
|  | (SU("Self-Inflicted Wounds" OR "suicidal behavior" OR "suicide" OR "youth suicide" OR "suicidality") OR (TI("suicid*" OR "self-kill*" OR "self-poison*" OR "self-stabb*" OR "parasuicid*" OR "self mutilat*" OR "selfmutilat*" OR "automutilat*" OR "auto mutilat*" OR "Self-Injur*" OR "Self Harm*" OR "Self-Destructive Behavio*" OR "Self cutting" OR "self directed violence" OR "self inflicted injur*" OR "self wounding" OR "self-inflicted harm" OR "self-inflicted mutilat*" OR "self-inflicted wound*" OR selfharm* OR "selfinflicted injur*" OR "selfinflicted wound*" OR "head banging")) OR (AB("suicid*" OR "self-kill*" OR "self-poison*" OR "self-stabb*" OR "parasuicid*" OR "self mutilat*" OR "selfmutilat*" OR "automutilat*" OR "auto mutilat*" OR "Self-Injur*" OR "Self Harm*" OR "Self-Destructive Behavio*" OR "Self cutting" OR "self directed violence" OR "self inflicted injur*" OR "self wounding" OR "self-inflicted harm" OR "self-inflicted mutilat*" OR "self-inflicted wound*" OR selfharm* OR "selfinflicted injur*" OR "selfinflicted wound*" OR "head banging")) |
| AND |  |
|  | (SU("Child Psychiatry" OR "Child Psychology" OR "Child Psychopathology" OR "Child Psychotherapy" OR "Pediatrics") OR (TI("child*" OR "preschool" or "pre-school" OR "infant" OR "pubescen*" OR "kid*" OR "minors" OR "paediatric*" OR "pediatric*" OR "youth*" OR "teenage*")) OR (AB("child*" OR "preschool" OR "pre-school" OR "infant" OR "pubescen*" OR "kid*" OR "minors" OR "paediatric*" OR "pediatric*" OR "youth*" OR "teenage*")) |
|  | OR |
|  | (SU("Adolescent Health" OR "Adolescent Psychiatry" OR "Adolescents Psychology" OR "Adolescent Psychopathology" OR "Adolescent Psychotherapy") OR (TI("Adolescen*" OR "Teen*" OR "Youth*" OR "young adult*" OR "young age" OR "young patient*" OR "young person*" OR "student*" OR "young people*" OR "youngsters")) OR (AB("Adolescen*" OR "Teen*" OR "Youth*" OR "young adult*" OR "young age" OR "young patient*" OR "young person*" OR "student*" OR "young people*" OR "youngsters")) |
|  | OR |
|  | (SU("Emerging Adulthood") OR (TI("student*" OR "freshman" OR "freshmen" OR "late adolesc*" OR "emerging adult*" OR "new adult*" OR "emerging adult*" OR "undergraduate student*" OR "graduate student*" OR "college student*")) OR (AB("student*" OR "freshman" OR "freshmen" OR "late adolesc*" OR "emerging adult*" OR "new adult*"OR "emerging adult*" OR "undergraduate student*" OR "graduate student*" OR "college student*")) |
| AND |  |
|  | (SU("Recovery (Disorders)" OR "Remission (Disorders)" OR "Treatment Outcomes" OR "Psychotherapeutic Outcomes" OR "Therapeutic Processes" OR "Resilience (Psychological)") OR (TI("Recover*" OR "Resilien*" OR "thriv*" OR "surviv*" OR "Treatment effect" OR "get better" OR reasons for living" or "overcome")) OR (AB("Recover*" OR "Resilien*" OR "thriv*" OR "surviv*" OR "Treatment effect" OR "get better" OR reasons for living" or "overcome")) |
| **SOCIndex** |  |
|  | (SU("SUICIDE" OR "SUICIDAL behavior" OR "SUICIDAL ideation" OR "SUICIDE – Psychological aspects" OR "Suicide – Social aspects" OR "SUICIDAL behavior in young adults" OR "SUICIDAL behavior in youth" OR "SUICIDAL behavior of children" OR "SUICIDAL behavior in college students") OR (TI("suicid*" OR "self-kill*" OR "self-poison*" OR "self-stabb*" OR "parasuicid*" OR "self mutilat*" OR "selfmutilat*" OR "automutilat*" OR "auto mutilat*" OR "Self-Injur*" OR "Self Harm*" OR "Self-Destructive Behavio*" OR "Self cutting" OR "self directed violence" OR "self inflicted injur*" OR "self wounding" OR "self-inflicted harm" OR "self-inflicted mutilat*" OR "self-inflicted wound*" OR selfharm* OR "selfinflicted injur*" OR "selfinflicted wound*" OR "head banging")) OR (AB("suicid*" OR "self-kill*" OR "self-poison*" OR "self-stabb*" OR "parasuicid*" OR "self mutilat*" OR "selfmutilat*" OR "automutilat*" OR "auto mutilat*" OR "Self-Injur*" OR "Self Harm*" OR "Self-Destructive Behavio*" OR "Self cutting" OR "self directed violence" OR "self inflicted injur*" OR "self wounding" OR "self-inflicted harm" OR "self-inflicted mutilat*" OR "self-inflicted wound*" OR selfharm* OR "selfinflicted injur*" OR "selfinflicted wound*" OR "head banging")) |
|  |  |
| AND | (SU("CHILDREN" OR "CHILD psychiatry" OR "CHILD psychology" OR "CHILD psychotherapy") OR (TI("child*" OR "preschool" OR "pre-school" OR "infant" OR "pubescen*" OR "kid*" OR "minors" OR "paediatric*" OR "pediatric*" OR "youth*" OR "teenage*")) OR (AB(child*" OR "preschool" OR "pre-school" OR "infant" OR "pubescen*" OR "kid*" OR "minors" OR "paediatric*" OR "pediatric*" OR "youth*" OR "teenage*")) |
|  | OR |
|  | (SU("ADOLESCENCE" OR "TEENAGERS" OR "ADOLESCENT psychology" OR "ADOLESCENT psychotherapy – evaluation") OR (TI("Adolescen*" OR "Teen*" OR "Youth*" OR "young adult*" OR "young age" OR "young patient*" OR "young person*" OR "student*" OR "young people*" OR "youngsters")) OR (AB("Adolescen*" OR "Teen*" OR "Youth*" OR "young adult*" OR "young age" OR "young patient*" OR "young person*" OR "student*" OR "young people*" OR "youngsters")) |
|  | OR |
|  | (SU("Emerging Adulthood") OR (TI("student*" OR "freshman" OR "freshmen" OR "late adolesc*" OR "emerging adult*" OR "new adult*"OR "emerging adult*" OR "undergraduate student*" OR "graduate student*" OR "college student*")) OR (AB("student*" OR "freshman" OR "freshmen" OR "late adolesc*" OR "emerging adult*" OR "new adult*" OR "emerging adult*" OR "undergraduate student*" OR "graduate student*" OR "college student*")) |
| AND |  |
|  | (SU("PSYCHOTHERAPY" OR "TERMINATING of psychotherapy" OR "PSYCHOTHERAPY practice" OR "PSYCHOTHERAPY – Social aspects") OR (TI("Recover*" OR "Resilien*" OR "thriv*" OR "surviv*" OR "Treatment effect" OR "get better" OR reasons for living" or "overcome")) OR (AB("Recover*" OR "Resilien*" OR "thriv*" OR "surviv*" OR "Treatment effect" OR "get better" OR reasons for living" or "overcome")) |
| **Medline** |  |
|  | (SU("Self-Injurious Behavior" OR "Suicide" OR "Self Mutilation" OR "Suicidal Ideation") OR (TI("suicid*" OR "self-kill*" OR "self-poison*" OR "self-stabb*" OR "parasuicid*" OR "self mutilat*" OR "selfmutilat*" OR "automutilat*" OR "auto mutilat*" OR "Self-Injur*" OR "Self Harm*" OR "Self-Destructive Behavio*" OR "Self cutting" OR "self directed violence" OR "self inflicted injur*" OR "self wounding" OR "self-inflicted harm" OR "self-inflicted mutilat*" OR "self-inflicted wound*" OR selfharm* OR "selfinflicted injur*" OR "selfinflicted wound*" OR "head banging")) OR (AB("suicid*" OR "self-kill*" OR "self-poison*" OR "self-stabb*" OR "parasuicid*" OR "self mutilat*" OR "selfmutilat*" OR "automutilat*" OR "auto mutilat*" OR "Self-Injur*" OR "Self Harm*" OR "Self-Destructive Behavio*" OR "Self cutting" OR "self directed violence" OR "self inflicted injur*" OR "self wounding" OR "self-inflicted harm" OR "self-inflicted mutilat*" OR "self-inflicted wound*" OR selfharm* OR "selfinflicted injur*" OR "selfinflicted wound*" OR "head banging")) |
| AND |  |
|  | (SU("Child" OR "Psychology, Child" OR "Child Psychiatry" OR "Child Health") OR (TI("child*" OR "preschool or pre-school" OR "infant" OR "pubescen*" OR "kid*" OR "minors" OR "paediatric*" OR "pediatric*" OR "youth*" OR "teenage*")) OR (AB("child*" OR "preschool or pre-school" OR "infant" OR "pubescen*" OR "kid*" OR "minors" OR "paediatric*" OR "pediatric*" OR "youth*" OR "teenage*")) |
|  | OR |
|  | (SU("Adolescent" OR "Adolescent Psychiatry" OR "Psychology, Adolescent") OR (TI("Adolescen*" OR "Teen*" OR "Youth*" OR "young adult*" OR "young age" OR "young patient*" OR "young person*" OR "student*" OR "young people*" OR "youngsters")) OR (AB("Adolescen*" OR "Teen*" OR "Youth*" OR "young adult*" OR "young age" OR "young patient*" OR "young person*" OR "student*" OR "young people*" OR "youngsters")) |
|  | OR |
|  | (SU("Young Adult") OR (TI("student*" OR "freshman" OR "freshmen" OR "late adolesc*" OR "emerging adult*" OR "new adult*"OR "emerging adult*" OR "undergraduate student*" OR "graduate student*" OR "college student*")) OR (AB("student*" OR "freshman" OR "freshmen" OR "late adolesc*" OR "emerging adult*" OR "new adult*" OR "emerging adult*" OR "undergraduate student*" OR "graduate student*" OR "college student*")) |
| AND |  |
|  | (SU("Mental Health Recovery" OR "Resilience, Psychology" OR "Treatment Outcome" OR "Psychotherapeutic Processes") OR (TI("Recover*" OR "Resilien*" OR "thriv*" OR "surviv*" OR "Treatment effect" OR "get better" OR reasons for living" or "overcome")) OR (AB("Recover*" OR "Resilien*" OR "thriv*" OR "surviv*" OR "Treatment effect" OR "get better" OR reasons for living" or "overcome")) |
| **CINAHL** |  |
|  | (SU("Suicide" OR "Suicide, Attempted" OR "Suicidal Ideation" OR "Self-injurious Behavior") OR (TI("suicid*" OR "self-kill*" OR "self-poison*" OR "self-stabb*" OR "parasuicid*" OR "self mutilat*" OR "selfmutilat*" OR "automutilat*" OR "auto mutilat*" OR "Self-Injur*" OR "Self Harm*" OR "Self-Destructive Behavio*" OR "Self cutting" OR "self directed violence" OR "self inflicted injur*" OR "self wounding" OR "self-inflicted harm" OR "self-inflicted mutilat*" OR "self-inflicted wound*" OR selfharm* OR "selfinflicted injur*" OR "selfinflicted wound*" OR "head banging")) OR (AB("suicid*" OR "self-kill*" OR "self-poison*" OR "self-stabb*" OR "parasuicid*" OR "self mutilat*" OR "selfmutilat*" OR "automutilat*" OR "auto mutilat*" OR "Self-Injur*" OR "Self Harm*" OR "Self-Destructive Behavio*" OR "Self cutting" OR "self directed violence" OR "self inflicted injur*" OR "self wounding" OR "self-inflicted harm" OR "self-inflicted mutilat*" OR "self-inflicted wound*" OR selfharm* OR "selfinflicted injur*" OR "selfinflicted wound*" OR "head banging")) |
| AND |  |
|  | (SU("Child" OR "Child Psychiatry" OR "Child Psychology" OR "Child Health") OR (TI("child*" OR "preschool or pre-school" OR "infant" OR "pubescen*" OR "kid*" OR "minors" OR "paediatric*" OR "pediatric*" OR "youth*" OR "teenage*")) OR (AB("child*" OR "preschool or pre-school" OR "infant" OR "pubescen*" OR "kid*" OR "minors" OR "paediatric*" OR "pediatric*" OR "youth*" OR "teenage*")) |
|  | OR |
|  | (SU("Adolescence") OR (TI("Adolescen*" OR "Teen*" OR "Youth*" OR "young adult*" OR "young age" OR "young patient*" OR "young person*" OR "student*" OR "young people*" OR "youngsters")) OR (AB("Adolescen*" OR "Teen*" OR "Youth*" OR "young adult*" OR "young age" OR "young patient*" OR "young person*" OR "student*" OR "young people*" OR "youngsters")) |
|  | OR |
|  | (SU("Young Adult") OR (TI("student*" OR "freshman" OR "freshmen" OR "late adolesc*" OR "emerging adult*" OR "new adult*"OR "emerging adult*" OR "undergraduate student*" OR "graduate student*" OR "college student*")) OR (AB("student*" OR "freshman" OR "freshmen" OR "late adolesc*" OR "emerging adult*" OR "new adult*" OR "emerging adult*" OR "undergraduate student*" OR "graduate student*" OR "college student*")) |
| AND |  |
|  | (SU("Hardiness" OR "Recovery" OR "Mental Health Treatment (Saba CCC)" OR "Treatment Outcomes") OR (TI("Recover*" OR "Resilien*" OR "thriv*" OR "surviv*" OR "Treatment effect" OR "get better" OR reasons for living" or "overcome")) OR (AB("Recover*" OR "Resilien*" OR "thriv*" OR "surviv*" OR "Treatment effect" OR "get better" OR reasons for living" or "overcome")) |

**Table S2. Quality assessment by CASP for qualitative studies.**

| **CASP Question** | **Aims stated clearly** | **Qualitative methodology appropriate** | **Research design appropriate to address aims of the research** | **Recruitment strategy appropriate to aims** | **Data collection method address the research issue** | **Relationship between researcher and participants considered** | **Ethical issues been taken into consideration** | **Data analysis rigorous** | **Clear statement of the finding** | **Research is valuable** |
| --- | --- | --- | --- | --- | --- | --- | --- | --- | --- | --- |
| **Authors (year)** |  |  |  |  |  |  |  |  |  |  |
| Bautista et al. (2023) | Adequate | Yes | Yes | Can't tell | Yes | Can't tell | Yes | Yes | Adequate | Yes |
| Bergmans et al. (2009) | Yes | Yes | Yes | Adequate | Yes | Can't tell | Yes | Yes | Yes | Yes |
| Bennett et al. (2002) | Yes | Yes | Yes | Yes | Yes | Can't tell | No | Yes | No | Yes |
| Bostik et al. (2007) | Yes | Yes | Yes | Yes | Yes | Can't tell | Yes | Yes | Yes | Yes |
| Buser et al. (2014) | Yes | Yes | Yes | Yes | Yes | Can't tell | Yes | Yes | Yes | Yes |
| Claréus et al. (2021) | Adequate | Yes | Yes | Adequate | No | Yes | Adequate | Yes | Yes | Yes |
| Everall et al. (2005) | Yes | Yes | Yes | No | Yes | Can't tell | Adequate | Yes | Yes | Adequate |
| Everall et al. (2006) | Adequate | Yes | Adequate | Adequate | Adequate | Can't tell | Yes | Adequate | Yes | Yes |
| Fayaz (2022) | Adequate | Yes | Yes | Can't tell | Can't tell | Can't tell | Can't tell | No | No | No |
| Fenaughty et al. (2003) | Adequate | Yes | Adequate | Can't tell | Adequate | Can't tell | Adequate | Yes | Yes | Yes |
| Gelinas et al. (2013) | Yes | Adequate | Adequate | Adequate | Adequate | Can't tell | Yes | Yes | Yes | Yes |
| Gulbas et al. (2019) | Yes | Yes | Yes | Yes | Yes | Can't tell | Yes | Yes | Yes | Yes |
| Holliday & Vandermause (2015) | Yes | Yes | Yes | Can't tell | Yes | Can't tell | Yes | Yes | Yes | Yes |
| Keefner et al. (2020) | Yes | Yes | Yes | Yes | Yes | Can't tell | Yes | Yes | Yes | Yes |
| Kelada et al. (2018b) | Yes | Adequate | Adequate | Adequate | Adequate | Can't tell | Yes | Yes | Yes | Yes |
| Knowles et al. (2022) | Yes | Yes | Yes | Adequate | Yes | Can't tell | Yes | Yes | Adequate | Yes |
| Kolar et al. (2012) | Adequate | Yes | Yes | Yes | Yes | Can't tell | Yes | Yes | Adequate | Adequate |
| Lewis et al. (2019) | Yes | Adequate | Yes | Adequate | Yes | Can't tell | Yes | Yes | Yes | Yes |
| Mehali et al. (2022) | Yes | Yes | Yes | Adequate | Yes | Can't tell | Yes | Yes | Yes | Yes |
| Norton (2011) | Adequate | Yes | Adequate | No | Yes | Yes | No | No | Adequate | Adequate |
| Örzen-Dursun (2023) | Yes | Yes | Yes | Yes | Yes | Can't tell | Yes | Yes | Yes | Yes |
| Puotiniemi et al. (2004) | Yes | Yes | Adequate | Adequate | Yes | Can't tell | Yes | Yes | Yes | Yes |
| Redmond (2020) | Yes | Yes | Yes | Yes | Yes | Yes | Yes | Yes | Yes | Yes |
| Rissanen et al. (2013) | Yes | Yes | Yes | Yes | Yes | No | Yes | Yes | Yes | Yes |
| Shaw (2006) | Yes | Yes | Yes | Yes | Yes | Can't tell | No | Yes | Yes | Yes |
| Szlyk (2021) | Yes | Yes | Adequate | No | Yes | Can't tell | Yes | Yes | Yes | Yes |
| Tampus-Siena et al. (2023) | Yes | Yes | Yes | Can't tell | Yes | Yes | Yes | Yes | Adequate | Yes |
| Tomicic et al. (2021) | Yes | Yes | Yes | Yes | Yes | Can't tell | Yes | Yes | Yes | Yes |
| Wadman et al. (2017) | Yes | Yes | Yes | Yes | Yes | Can't tell | Yes | Yes | Yes | Yes |
| Wang et al. (2024) | Yes | Yes | Yes | Can’t tell | Yes | Can’t tell | Yes | Adequate | Yes | Yes |
| Whitlock et al. (2015) | Yes | Adequate | Yes | Yes | Yes | Can't tell | Yes | Yes | Yes | Yes |
| Zortea et al. (2019) | Yes | Yes | Yes | Yes | Yes | Yes | Yes | Yes | Yes | Yes |
| *PhD Dissertations* |  |  |  |  |  |  |  |  |  |  |
| Davis (2018) | Adequate | Yes | Yes | Yes | Yes | Yes | Yes | Yes | Yes | Yes |
| Gelinas (2015) | Yes | Yes | Yes | Adequate | Yes | Can't tell | Yes | Yes | Yes | Yes |
| Roberts (2019) | Yes | Yes | Yes | Can't tell | Yes | Can't tell | Adequate | Yes | Yes | Yes |

**Table S3. Quality assessment by QUIPS for quantitative studies.**

| **Authors (year)** | **Study participation** | **Study Attrition** | **Outcome Measurement** | **PF Measurement** | **Study Confounding** | **Statistical Analysis and Presentation** |
| --- | --- | --- | --- | --- | --- | --- |
| Buelens et al. (2023) | Low | Low | Low | Low | Low | Low |
| Clareus et al. (2023) | Low | Moderate | Low | Low | Low | Low |
| Goncalves et al. (2023) | Low | N/A | Low | Low | Low | Low |
| Hasking et al. (2024) | Moderate | N/A | Low | Low | N/A | Low |
| Kelada et al. (2018a) | Low | N/A | Low | Low | Low | Low |
| Kwok et al. (2019) | Low | Low | Low | Low | Low | Low |
| Meheli et al. (2023) | Low | N/A | Low | Moderate | High | Low |
| Muehlenkamp et al. (2019) | Low | N/A | Low | Low | Low | Low |
| Ortin et al. (2019) | Low | Low | Low | Low | Low | Low |
| Real et al. (2025) | Low | Moderate | Low | Low | Low | Low |
| Teismann et al. (2016) | Low | High | Moderate | Low | Low | Low |
| Tong et al. (2022) | Low | Low | Low | Low | Low | Low |
| Tordoff et al. (2022) | Low | High | Low | Low | Low | Low |
| Wagner et al. (2000) | Low | High | Low | Low | Low | Low |
| Wang et al. (2023) | Low | Low | Low | Moderate | Moderate | Low |
| Wiglesworth et al. (2023) | High | Low | Low | Low | Low | Low |
| Zeller et al. (2015) | Low | Moderate | Low | Low | High | Low |

**Table S4. List of all codes used.**

| **Code** | **# times coded** |
| --- | --- |
| Accepting environment | 19 |
| Accepting help | 7 |
| Access to formal support | 2 |
| Action by others | 1 |
| Addictiveness of behaviors | 4 |
| Age | 2 |
| Agency and control | 12 |
| Asking for help | 11 |
| Assessment | 2 |
| Attributing skills to personal ability | 1 |
| Attributional style | 1 |
| Awareness | 11 |
| Awareness of choice (autonomy) | 2 |
| Change in environment | 15 |
| Comorbidity | 2 |
| Compensating for past failures | 1 |
| Connection | 16 |
| Contributing to lives of others | 12 |
| Coping skills in general | 23 |
| Crisis management and safety planning | 2 |
| Desire for wellness | 6 |
| Diagnosis | 3 |
| Disapproval by others | 3 |
| Disclosure | 2 |
| Dissociation | 1 |
| Distraction | 14 |
| Emotion regulation | 19 |
| Emotional support | 2 |
| Empathy | 1 |
| Encouragement by others | 3 |
| Family functioning | 1 |
| Fear of death | 1 |
| Feeling heard / listened to | 7 |
| Formal support | 38 |
| Future and goal-directed action | 12 |
| Gender | 2 |
| Gratitude | 5 |
| Greater awareness about issues by others | 8 |
| Hope | 7 |
| Housing | 2 |
| Identity development | 1 |
| Informal support | 45 |
| Integration of behaviors | 1 |
| Internalizing behavior as personality | 1 |
| Internalizing support is there | 1 |
| Intervention | 1 |
| Less need to continue | 4 |
| Life event | 4 |
| Life satisfaction | 1 |
| Maturation | 3 |
| Meaning in life | 2 |
| Meaninglessness of behavior | 4 |
| Means restriction | 1 |
| Medication | 5 |
| Motherhood | 6 |
| Negative coping skills | 1 |
| No strength to continue | 1 |
| Non-specific aspects of treatment | 4 |
| Not wanting to hurt others | 12 |
| Older age of onset | 2 |
| Puberty blockers and gender-affirming hormones | 1 |
| Peer support | 8 |
| Perspective in general | 8 |
| Planning | 0 |
| Positive life events | 2 |
| Positive thinking | 3 |
| Practical costs of SH | 15 |
| Practical support in general | 6 |
| Previous STBs | 2 |
| Psycho-education | 7 |
| Rationalization | 1 |
| Reasons for living | 1 |
| Religion | 11 |
| Resilience | 3 |
| Resolving issues | 2 |
| School | 3 |
| Self-acceptance | 15 |
| Self-appraisal | 2 |
| Self-compassion | 3 |
| self-efficacy | 1 |
| Self-empathy | 1 |
| Self-esteem | 3 |
| Self-understanding | 5 |
| Self-worth | 5 |
| Service user freedom | 1 |
| Service user involvement / copartnership | 2 |
| Social activities (increase) | 1 |
| Something is wrong with me | 1 |
| Staff responsibility | 1 |
| Stopped by others during behavior | 1 |
| Stress | 3 |
| Symptom reduction and management | 7 |
| Talking | 7 |
| Unconditional support | 3 |
| Wellbeing | 2 |
|  |  |
|  |  |
| Barriers to recovery | 23 |
| Definition recovery | 24 |
